# Food‐Related Attentional Biases in Restrained Eaters: A Meta‐Analysis

**DOI:** 10.1002/eat.70090

**Published:** 2026-03-31

**Authors:** Rio Madan, Cristina Martinelli

**Affiliations:** ^1^ City St George's University of London London UK; ^2^ Kingston University London UK

**Keywords:** attentional biases, dietary restraint, disordered eating, eating disorders, eye‐tracking, food stimuli, meta‐analysis, response task

## Abstract

**Objective:**

Dietary restraint may contribute to the development and maintenance of eating disorders (EDs), with food‐related attentional biases (ABs) as a key underlying mechanism. We examined associations between dietary restraint and ABs and explored how several methodological factors (i.e., AB mechanism, mode of AB investigation, response task type, stimulus task relevance, and type of food stimulus) might influence these associations.

**Method:**

Database searches followed the guidelines set by the Preferred Reporting Items for Systematic Review and Meta‐analyses (PRISMA). We included empirical studies that measured both dietary restraint and ABs, excluding studies involving participants with clinical diagnoses or below 16 years of age. Fifty‐one eligible articles were identified, of which 29 unique samples were included in the final analyses. The protocol for this meta‐analysis was preregistered at: https://www.crd.york.ac.uk/prospero/display_record.php?RecordID=532562.

**Results:**

We first examined associations between dietary restraint and attentional maintenance and orienting, separately. This was followed by subgroup analyses to examine whether these associations varied based on the chosen methodological factors. Our findings revealed significant associations between dietary restraint and attentional maintenance in studies that used response tasks (other than the dot probe task), and where the food stimuli were relevant to the task instructions.

**Discussion:**

Collectively, these findings suggest that dietary restrainers activated strategic top‐down processing of food cues, rather than the reflexive orienting linked to ED‐driven saliency processing. Overall, this may be interpreted as more purposeful monitoring to facilitate restraint when food is relevant to the goals and actions of dietary restrainers.

## Summary

- Dietary restraint may contribute to the development and maintenance of eating disorders, with food‐related attentional biases as a key underlying mechanism.
- Current literature on food‐related attentional biases and dietary restraint is contradictory.
- Different methodological approaches used to capture attentional biases may account for some of this divergence.
- Our meta‐analysis revealed that associations between dietary restraint and attentional biases varied based on the specific attentional bias investigated, the mode of AB investigation, the response task type, and the relevance of food stimuli to the task.

## 1 Introduction

Dietary restraint involves exercising cognitive control to override physiological and environmental cues that signal food intake (Polivy and Herman 2020). With the abundance of ultra‐processed and high energy dense food promoting overeating and hedonic eating styles (Calcaterra et al. 2023; LaFata et al. 2024), dietary restraint is now a prevalent tool for weight loss or maintenance across cultures (Olea López and Johnson 2016; Alqahtani and Alhazmi 2025; Yong et al. 2021).

However, Restraint Theory argues that repeatedly exercising cognitive control over food intake can have a rebound effect, leading to increased preoccupation with food and greater susceptibility to external food cues (Herman and Polivy 1975). This can result in lapses in dietary control, which may trigger counterregulatory eating (Waliłko et al. 2021) and other maladaptive eating tendencies (Meule, Papies, and Kübler 2012; Meule, Vögele, and Kübler 2012), thereby contributing to disordered eating. Consistent with this, dietary restrainers are more likely to be overweight (Ramírez‐Contreras et al. 2021), experience more episodes of disinhibited eating (Waliłko et al. 2021) and binge eating (Linardon et al. 2020), have negative thinking patterns around food (Waliłko et al. 2021), and report more body dissatisfaction (Kong et al. 2013) than their unrestrained counterparts. Furthermore, dietary restraint has been shown to be a robust predictor and risk factor for clinical eating disorders (EDs; Racine et al. 2011; Yamamiya and Stice 2024).

Mechanisms thought to underlie food preoccupation and susceptibility to overeating in dietary restrainers relate to the cognitive processing of food‐related cues. Particularly, attentional biases (ABs)—defined as the selective allocation of attention towards specific stimuli whilst ignoring others—towards food have been observed in dietary restrainers (e.g., Hepworth et al. 2010; Veenstra et al. 2010; Dondzilo et al. 2022; Chen et al. 2023). It has been proposed that such ABs could drive the rebounding effect of dietary restraint and contribute to maladaptive eating behaviors (Polivy and Herman 2020). Consistent with this, food‐related ABs have been linked to higher cravings (Hardman et al. 2021), greater food intake (Hardman et al. 2021; Werthmann et al. 2014), higher BMI (Brand et al. 2024), increased disinhibited (Seage and Lee 2017), and external eating (Hou et al. 2011).

In clinical ED groups, increased attention to food cues has been linked to binge‐eating disorder (Schmidt et al. 2016; Deluchi et al. 2017; Stott et al. 2021), bulimia nervosa (Stott et al. 2021; Albery et al. 2016), and food addiction (Liu et al. 2025). In anorexia nervosa, where refusal to eat an adequate intake is the hallmark of the illness, ABs are characterized by an initial attentional orienting towards food cues—reflecting heightened threat detection (Neimeijer et al. 2017)—followed by attentional avoidance of food cues (Meregalli et al. 2023)—thought to reflect top‐down goal‐driven processing that maintains severe energy restriction.

However, the current evidence for food‐related ABs in dietary restrainers is equivocal, and the link to disordered eating remains largely unexplored. This gap is important to address as ABs towards food stimuli are known to contribute to EDs in clinical populations (Mercado et al. 2020; Flynn 2023; Werle et al. 2024) and may also play a role in maladaptive eating among dietary restrainers (Waliłko et al. 2021). Thus, it remains unclear whether food‐related ABs exist and contribute to ED risk in non‐clinical yet vulnerable groups such as dietary restrainers. To date, only one study has attempted a systematic review of the literature. Watson and Le Pelley (2021) examined associations between dietary restraint and several cognitive biases but reported inconclusive results regarding the relationship between dietary restraint and ABs. Furthermore, the review did not investigate links between dietary restraint and disordered eating.

We here propose that the inconsistent findings in the literature on associations between ABs and dietary restraint may be attributable, at least in part, to heterogeneous methodological approaches. One major source of this heterogeneity lies in the different types of ABs that have been examined. The literature typically distinguishes between attentional maintenance (van Ens et al. 2019), attentional orienting (van Ens et al. 2019), distraction (Neimeijer et al. 2013), interference (Neimeijer et al. 2013), switching (Dondzilo et al. 2022), and disengagement (Veenstra et al. 2010) processes, each assessed using varying methodological approaches. A second source of heterogeneity relates to the measurement of ABs. Studies have employed both reaction time (RT) based tasks (Ahern et al. 2010; Meule, Papies, and Kübler 2012; Meule, Vögele, and Kübler 2012) and eye‐tracking methods, with the latter further subdivided into instructed (Liu et al. 2021; Werthmann, Roefs, Nederkoorn, and Jansen 2013; Werthmann, Roefs, Nederkoorn, Mogg, et al. 2013) and free‐viewing paradigms (Hummel et al. 2018; Graham et al. 2011). Additionally, tasks vary in the food‐related stimuli used (Dondzilo et al. 2022; Brignell et al. 2009) and in whether these stimuli are relevant to task instructions (Donofry et al. 2019; Jiang et al. 2024).

To address these issues, we conducted a series of meta‐analyses examining how these methodological factors influence the relationship between food‐related ABs and dietary restraint. Our work builds on the review by Watson and Le Pelley (2021) by (1) focusing exclusively on ABs, rather than a broader range of cognitive biases, (2) including eye‐tracking data, which were largely overlooked in their work, (3) isolating distinct AB mechanisms that were not differentiated in their analyses, (4) investigating specific methodological factors not previously considered, and (5) exploring associations between ABs and ED symptoms. In addition, we further reduced heterogeneity present in their investigation by restricting inclusion to studies using pictorial stimuli only (rather than combining pictorial and linguistic stimuli), by excluding studies where main findings were based on experimental manipulations of mood or hunger which may have biased results, and controlling (where possible) for whether studies screened out clinical EDs. This led to the inclusion of 8 samples from Watson and Le Pelley's (2021) review and 21 new samples they did not include. Overall, our goal was to determine whether dietary associations between dietary restraint and ABs are tied to specific methodological factors, and whether such associations are also related to disordered eating in undiagnosed, yet ED‐vulnerable populations, such as dietary restrainers.

### 1.1 Research Questions

1. Does the relationship between ABs and dietary restraint vary as a function of attentional mechanisms (i.e., orienting vs. maintenance), modes of AB investigation (i.e., eye‐tracking vs. response tasks; free‐viewing vs. instructed‐viewing eye‐tracking tasks), the response task used (i.e., dot probe vs. other tasks), the relevance of food stimuli to task instructions (i.e., task relevant vs. task irrelevant), and type of food stimuli (i.e., high calorie [HC] vs. mixed foods)?
2. Is the association between dietary restraint and ABs linked to disordered eating?

Research question 1 focuses on potential sources of heterogeneity in the association between dietary restraint and ABs. Given the methodological diversity of AB paradigms and evidence that different tasks and attentional indices capture distinct cognitive processes, it is important to examine whether observed associations vary systematically across attentional mechanisms, task characteristics, and stimulus properties. On the basis of the literature we hypothesized that stronger associations between restraint and ABs would be found for (i) eye‐tracking versus response tasks, as gaze indices have been found to be more reliable in inferring attentional allocation (Waechter et al. 2014); (ii) irrelevant versus relevant task stimuli, as irrelevant stimuli have been shown to carry more bottom‐up saliency (Neimeijer et al. 2017) and to easily elicit ABs in clinical populations (Sablottny et al. 2025; Neimeijer et al. 2017; Meregalli et al. 2023)—we predicted dietary restrainers to behave similarly; (iii) HC versus mixed foods, as HC cues may carry more reward‐ or threat‐related saliency for dietary restrainers (Wang et al. 2016); (iv) dot‐probe tasks versus alternative tasks, given the task homogeneity of the dot‐probe group.

## 2 Method

### 2.1 Search Strategy

Literature searches were guided by the Preferred Reporting Items for Systematic Review and Meta‐analyses (PRISMA; Page, McKenzie, et al. 2021; Page, Sterne, et al. 2021). Our search was conducted on April 2024 (updated on October 2024, July 2025 and December 2025) on Web of Science, PsycINFO, PubMed, CINAHL Plus, and Medline. In addition, we searched the references of all selected articles and relevant published reviews. Gray literature and unpublished articles were searched via ProQuest Dissertations, SocArXiv Papers, Web of Science and Medline. These were included to account for potential publication bias (Hopewell et al. 2007). The search terms for each database consulted can be found in the Supporting Information. For each concept, relevant search terms were chosen, guided by the literature, as well as the index terms and synonyms in database thesauri. The protocol for this meta‐analysis was preregistered at: https://www.crd.york.ac.uk/prospero/display_record.php?RecordID=532562. Note that contrary to what was reported in the protocol, we were unable to examine dietary restraint in relation to approach versus avoidance biases due to insufficient studies measuring such biases. We were also unable to proceed with secondary study aims due to insufficient studies measuring any one type of experimental manipulation and insufficient studies measuring ED symptoms in the high dietary restraint group. Instead, we conducted an exploratory analysis of ED symptoms and AB associations unstratified by restraint. Finally, we conducted a univariate rather than a multivariate meta‐analysis due to the high degree of heterogeneity across studies and the risk that aggregating multiple correlated outcomes into composite effect sizes could mask distinct effects (Borenstein et al. 2021; please see Supporting Information).

### 2.2 Eligibility Criteria

To be included, studies had to (i) be empirical in nature, (ii) use a validated measure of dietary restraint (self‐reported or interviewed), and (iii) assess ABs to pictorial or video food stimuli. We included food stimuli of unspecified caloric value as well as food stimuli from a specific caloric category (high or low). Linguistic stimuli were excluded because they may be less ecologically valid for food‐specific investigations (Freijy et al. 2014), and their inclusion would have increased heterogeneity, as ABs are known to differ across stimulus modalities (Freijy et al. 2014; Stormark and Torkildsen 2004). To capture ABs in dietary restrainers, a population known to be at risk for EDs, we excluded studies involving clinical samples. We also excluded samples below 16 years of age, as feeding practices are largely shaped by parental influence in childhood and early adolescence (van Strien and Bazelier 2007), and dieting behaviors typically emerge in middle to late adolescence (Field et al. 2003). Reviews, meta‐analyses, qualitative studies, and study protocols were omitted. We excluded articles which assessed ABs after any experimental induction but included control group data if this was collected prior to manipulation. We also excluded data from any collapsed experimental and control conditions, to avoid food‐related ABs being confounded by specific manipulations such as those linked to negative mood (Hepworth et al. 2010) and hunger (Forestell et al. 2012). It was agreed to exclude studies using inhibitory control tasks due to a lack of consensus on whether these tasks can isolate ABs (Kakoschke et al. 2015). We also excluded tasks measuring any other type of cognitive bias, such as memory bias or impulsivity. Our primary outcome data were statistical associations between dietary restraint and ABs.

### 2.3 Data Selection and Extraction

**FIGURE 1:**
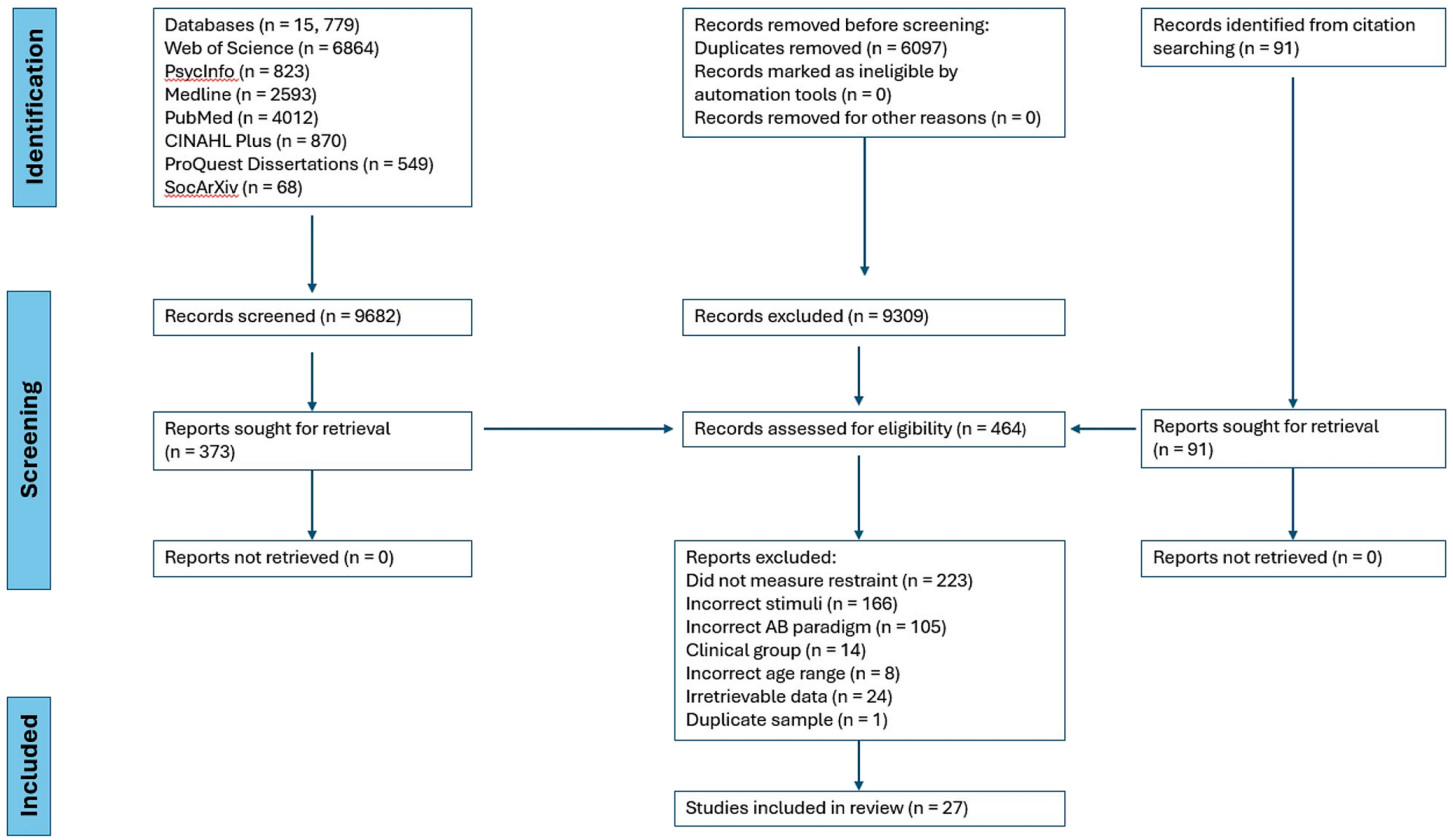
PRISMA flow diagram of study inclusion.

The study inclusion process is shown in the PRISMA Flow Diagram (Page, McKenzie, et al. 2021; Page, Sterne, et al. 2021) in Figure 1. Searches were conducted by one researcher. All retrieved studies were uploaded to Rayyan and duplicate studies were removed, leaving 9682 studies for screening. Two researchers independently screened studies, first by title and abstract and then by full text. Disagreements were resolved upon discussion. The results of a Cohen's Kappa test revealed good inclusion agreement between researchers (*k* = 0.720, *p* < 0.001; Landis and Koch 1977). This database search resulted in 36 eligible articles. The screening of 24 reviews on attentional and cognitive biases, and eye‐tracking research, in general and ED populations, yielded another 12 eligible studies. An additional 3 studies were identified by searching the references of all studies included. A final citation search of these three studies was carried out with no eligible studies identified.

Of the 51 total studies, 27 provided the quantitative data needed for the meta‐analyses, for some or all reported outcomes. Of these 27 studies, 11 authors provided this data via email correspondence (Nannt et al. 2025; Xu et al. 2023; Meule, Papies, and Kübler 2012; Meule, Vögele, and Kübler 2012; Sambal et al. 2021; Chen et al. 2023; Werthmann, Roefs, Nederkoorn, and Jansen 2013; Werthmann, Roefs, Nederkoorn, Mogg, et al. 2013; Werthmann et al. 2014; Garcia‐Burgos et al. 2017; Wilson and Wallis 2013; Kim et al. 2014; Hummel et al. 2018; Donofry et al. 2019). Of the 51 total studies, 24 had missing data required for the meta‐analyses (i.e., did not report stats on the association between AB and dietary restraint) that could not be retrieved from email correspondence—authors were contacted up to four times over a 5‐month period (Soetens et al. 2008; Burmester et al. 2022; Jonker et al. 2019; Kochs et al. 2022; Loeber et al. 2012, 2013; Stamataki et al. 2020; Valenzuela 2019; Seo and Lee 2021; Seage and Lee 2017; Hardman et al. 2014; Broadley et al. 2019; Baldofski et al. 2018; Hege et al. 2017; Geyskens et al. 2010; Smeets et al. 2009; Hou et al. 2011; Leppanen et al. 2017; Gearhardt et al. 2012; Higgs et al. 2012; Frayn et al. 2016; Sperling et al. 2017; Bongers et al. 2015; Hummel et al. 2017). As Wilson and Wallis (2013) contributed three separate samples, this resulted in a total of 29 included samples across 27 articles.

We extracted the following study and sample characteristics: year of publication, sample size, recruitment setting and country, race, ethnicity, socioeconomic status, gender, mean age, mean BMI, dietary restraint scale used, ED measures and whether participants were screened for EDs. Task‐related information was also extracted, including food type (e.g., HC food), comparator stimuli, stimuli relevance to task instructions, mode of AB investigation (response task vs. eye‐tracking), task type used, and, for eye‐tracking studies, whether paradigms involved free‐ or instructed‐viewing. We also extracted information on the AB mechanism investigated, the type of index used, and the relevant computation method. Extracted statistical data included *F*‐values or correlations between dietary restraint and ABs, means and standard deviations of the AB outcomes (separately for high restraint and low/no restraint in group comparison studies), mean ED scores and correlations between ABs and ED symptoms. Data collection took place between 2024 and 2025.

### 2.4 Effect Size Selection Criteria

We used the following criteria to select one effect size per study (see Supporting Information for selected effect sizes):

- When studies examined HC and low calorie (LC) food stimuli separately, HC outcomes were prioritized as being the most common stimuli among our studies. If studies compared HC food stimuli to both LC and non‐food stimuli, we prioritized LC comparator outcomes, as these capture a more nuanced effect of energy density, which is relevant to dietary restraint.
- If multiple modes of AB investigation were used, we used the following order of selection: free‐viewing gaze indices, instructed‐viewing gaze indices, and response task indices. This order reflects evidence that gaze patterns more directly index attentional allocation and demonstrate greater internal reliability compared to response tasks (Duc et al. 2008), and that free‐viewing paradigms provide greater ecological validity than instructed viewing tasks (Puttevils et al. 2023; Pasqualette and Kulke 2024).

#### 2.4.1 Maintenance Criteria—Primary Analysis

- Outcomes from trials of 500 ms or more; shifts in attention can occur as early as 500 ms (Field and Cox 2008). If more than one trial presentation time at 500 ms or more was investigated, the longer trial outcome was chosen, as these are deemed to be more reliable in capturing maintained attention (Skinner et al. 2018).
- Eye‐tracking indices reflecting maintenance: total dwell time or total fixation duration. If both indices were investigated, we prioritized total dwell time, being the most common in our studies. No current evidence suggests one is more reliable than the other.

#### 2.4.2 Orienting Criteria—Exploratory Analysis

- Outcomes from trials of 200 ms or less, as this timeframe is thought to capture initial orienting (Field and Cox 2008).
- Eye‐tracking indices typically used to measure orienting: gaze direction bias or first fixation location. If both indices were investigated, we prioritized first fixation location with previous evidence supporting good reliability (Soleymani et al. 2022) versus gaze direction bias (van Ens et al. 2019).

#### 2.4.3 ED Symptoms Criteria—Exploratory Analysis

- We prioritized attentional maintenance being the most common outcome investigated in relation to ED symptoms.
- If more than one ED outcome measure was used, we prioritized general over specific symptom measures, being the most common in included studies.

### 2.5 Coding of Data

We coded the data so that positive effect sizes reflected a stronger AB towards food stimuli (vs. comparator stimuli) as dietary restraint increased or in the high dietary restraint group. Negative effect sizes reflected a stronger AB towards food stimuli (vs. comparator stimuli) as dietary restraint decreased or in the low dietary restraint group. When there was no inclusion of a comparator stimulus, we included associations between restraint and response task data or gaze data for the food stimuli only (Jiang et al. 2024; Husted et al. 2016). For one study (Donofry et al. 2019), we recoded the AB score of food minus instrument trials so that a positive AB score reflected a stronger bias towards food versus non‐food.

### 2.6 Risk of Bias

The risk‐of‐bias assessment was carried out by two independent assessors. The Newcastle‐Ottawa quality assessment scale (NOS; Wells et al. 2000) was adapted to encompass factors relevant to our study, such as sample representativeness, the exclusion of conditions affecting eating behaviors, validity of the restraint outcome measure, sample size justification, the control of confounding variables, paradigm conditions, practice effects and randomization, and statistical analysis and reporting of results (see Supporting Information). Studies could score in the following categories: unsatisfactory (0–6 points), satisfactory (7–9 points), good (10–12 points), or very good (13–14 points). Discrepancies between scores in each of these domains were addressed by a third independent assessor.

### 2.7 Data Analysis

To account for smaller sample sizes, we selected Hedge's *G* as our standardized effect size (Taylor and Alanazi 2023). We first explored ROB using meta‐regression to determine whether to include high ROB studies in our analyses. ROB was added as an independent variable and associations between dietary restraint and ABs were the dependent variable. If no significant association was found, we included all studies in our analyses (Page, McKenzie, et al. 2021; Page, Sterne, et al. 2021).

We conducted univariate analyses to explore each of our research questions. Mode of AB investigation was explored in two ways: (1) with two subgroups, comparing response tasks to all viewing paradigms (instructed and free‐viewing) and (2) with three subgroups, comparing response tasks, instructed‐viewing, and free‐viewing. Type of response task had two subgroups comparing the dot probe task to all other tasks collectively, due to the low study number in each alternative task. Free‐viewing paradigms were excluded from this analysis as no specific task is used. Stimuli task relevance also had two subgroups: task relevant and task irrelevant stimuli, and again we excluded free‐viewing paradigms from this analysis as they do not include specific task instructions. Type of stimuli included two subgroups: mixed food and HC food. Finally, associations between ABs and ED symptoms were explored both collectively and across three subgroups of outcome measures used: Eating Disorder Examination Questionnaire (EDE‐Q), Eating Disorder Inventory (EDI‐2), and all other outcome measures collectively. For all subgroup analyses, we used meta‐regression models to adjust for potential confounders. These were whether clinical EDs were screened out and the type of restraint scale used. We did not run meta‐regression analyses if heterogeneity within subgroups was high, as this level of variability among studies can obscure true effects and lead to incorrect conclusions (Zhou and Shen 2022). We chose random effects models for all analyses to account for unexplained heterogeneity among effect sizes, and we used the following control variables in meta‐regression models: (1) whether clinical EDs were screened out; and (2) the type of restraint scale used. In line with Cochrane guidelines, we did not include control variables in meta‐regressions if heterogeneity was already low in the unadjusted models (Chandler et al. 2019). We used the *I*‐squared statistic and the following cut‐offs to assess heterogeneity: Low: 25%, Moderate: 50%, and High: 75% (Higgins et al. 2003).

Attentional maintenance outcomes were examined in the primary analysis, as this was the most frequently reported AB in the included studies. Attentional orienting outcomes were examined in a secondary exploratory analysis, as this was the second most commonly reported AB. There were insufficient studies reporting on other types of ABs to warrant further meta‐analyses.

## 3 Results

The 29 included samples had a total of 1727 participants, who were mainly adult females, mostly recruited via UK and European‐based universities. Their mean age range was 19–34 years, and most reported a healthy BMI. Table 1 shows the information extracted from all studies included in the meta‐analysis and our ROB final score.

**TABLE 1:** Study characteristics.

Study	Sample size	Gender	Mean age	Mean BMI	Sample and recruitment					Screening and restraint	
					Ethnicity	Race	SES	Country	Recruitment location	ED screened	Restraint scale
Nannt et al. (2025)	37	Female	31	22	NR	NR	NR	DE	University and Community	Yes	EDEQ
Liu et al. (2019)	56	Female	19	24	NR	NR	NR	NR	University, students	No	RS
Liu et al. (2021)	85	Female	21	22	NR	NR	NR	NL	Community and university	No	RS
Garcia‐Burgos et al. (2017)	46	Mixed	22	22	NR	NR	NR	CH	Students	Yes	DEBQ
Meule, Papies, and Kübler (2012) and Meule, Vögele, and Kübler (2012)	47	Female	23	RE = 23 URE = 21	NR	NR	NR	DE	University, students	No	RS
Dondzilo et al. (2022)	90	Mixed	20	RE = 24 URE = 20	NR	NR	NR	AU	University, students	No	RS
Werthmann, Roefs, Nederkoorn, and Jansen (2013)	45	Female	22	RE = 22 URE = 21	NR	NR	NR	NR	NR	No	RS
Werthmann, Roefs, Nederkoorn, Mogg, et al. (2013) (desire)	20	Female	NR	22	NR	NR	NR	NR	University, students	No	RS
Werthmann et al. (2014)	43	Female	21	22	NR	NR	NR	NR	Students from flyers, Facebook and email	No	RS
Wilson and Wallis (2013)	60	Female	20	RE = 24 URE = 22	NR	NR	NR	UK	University and community	Yes	DEBQ
Wilson and Wallis (2013)	58	Female	21	RE = 23 URE = 21	NR	NR	NR	UK	University and community	Yes	DEBQ
Wilson and Wallis (2013)	35	Female	21	NE = 23	NR	NR	NR	UK	University and community	Yes	DEBQ
Freijy et al. (2014)	99	Mixed	19	22	NR	Caucasian (54%)	Lives with parent (65%)	AU	Students, university	No	DEBQ
Veenstra et al. (2010)	28	Female	NR	RE = 25 URE = 21	NR	NR	NR	NL	Students, university	No	DEBQ
Neimeijer et al. (2013)	80	Female	21	RE = 23 URE = 21	NR	NR	NR	NL	Students, university	No	RS
Jiang et al. (2024)	52	Mixed	20	HE	NR	NR	NR	NR	Students, university	Yes	DEBQ
Sambal et al. (2021)	40	Female	24	24	NR	NR	NR	IL	Students, university	Yes	DEBQ
Donofry et al. (2019)	50	Female	21	23	NR	NR	NR	NL	Students, university	No	RS
Chen et al. (2023)	65	Mixed	19	RE = 20 URE = 20	NR	NR	NR	CN	NR	No	DEBQ
Hummel et al. (2018)	49	Mixed	23	23	NR	NR	NR	DE	University, students	No	DEBQ
Xu et al. (2023)	214	Mixed	20	23	NR	NR	NR	CA	University, “primarily students”	No	RS
Graham et al. (2011)	15	Female	23	29	Hispanic (30.6%)	Caucasian (55.6%); African American (2.8%); Other (2.8%)	NR	USA	Students, university	No	RS
Ahern et al. (2010)	63	Female	20	23	NR	Caucasian (79%); Asian (13%); Other (8%)	NR	NR	Students, university	No	DEBQ
Brignell et al. (2009)	53	Mixed	34	28	NR	NR	EDU: Sch (9.3%); Col (18.6%); Uni (72.1%)	UK	University and community	No	DEBQ
Hardman et al. (2013)	60	Mixed	23	22	NR	NR	NR	UK	Students, university	No	TFEQ
Kim et al. (2014)	34	Female	22	21	NR	NR	NR	KR	University, students	Yes	EDEQ
Husted et al. (2016)	47	Mixed	20	23	NR	NR	NR	UK	University and community	Yes	DEBQ
Kirsten et al. (2019)	103	Mixed	21	22	NR	NR	NR	DE	University students and community	Yes	DEBQ
van Ens et al. (2019)	53	Female	26	22	NR	NR	NR	UK	University and community	Yes	RS

Study	Attentional mechanism	Paradigm features					Standardized effect size and significance		Risk of bias
		Mode of AB investigation	Type of response task	Stimuli relevance	Target stimuli	Contrast stimuli	Hedge's *G*	*p*	
Nannt et al. (2025)	Maintenance	Instructed Viewing	DP	Irrelevant	HC	Non‐food	−0.382	0.264	G
Liu et al. (2019)	Maintenance	Response task	DP	Irrelevant	HC	Non‐food	0.320	0.243	S
Liu et al. (2021)	Maintenance	Instructed viewing	DP	Irrelevant	HC	Non‐food	−0.081	0.712	S
Garcia‐Burgos et al. (2017)	Maintenance	Free‐viewing	N/A		HC	LC	0.591	0.059	G
Meule, Papies, and Kübler (2012) and Meule, Vögele, and Kübler (2012)	Maintenance	Response task	FL	Relevant	HC	Non‐food	0.759	0.011	U
Dondzilo et al. (2022)	Maintenance	Response task	CF	Relevant	HC	LC	0.408	0.060	S
Werthmann, Roefs, Nederkoorn, and Jansen (2013)	Maintenance	Instructed‐viewing	DP	Irrelevant	HC	Non‐food	−0.144	0.623	U
Werthmann, Roefs, Nederkoorn, Mogg, et al. (2013) (desire)	Maintenance	Instructed viewing	DP	Irrelevant	HC	Non‐food	−0.328	0.486	U
Werthmann et al. (2014)	Maintenance	Instructed viewing	DP	Irrelevant	HC	Non‐food	−0.075	0.809	U
Wilson and Wallis (2013)	Maintenance	Response task	DP	Irrelevant	HC	Non‐food	0.047	0.856	U
Wilson and Wallis (2013)	Maintenance	Response task	DP	Irrelevant	HC	Non‐food	−0.236	0.377	S
Wilson and Wallis (2013)	Maintenance	Response task	DP	Irrelevant	HC	Non‐food	0.068	0.878	S
Freijy et al. (2014)	Maintenance	Response task	DP	Irrelevant	HC	Non‐food	0.020	0.921	S
Veenstra et al. (2010)	Maintenance	Response task	SC	Irrelevant	HC	Non‐food	0.235	0.548	S
Neimeijer et al. (2013)	Orienting	Response task	RSVP	Relevant	HC	Non‐food	0.135	0.542	S
Jiang et al. (2024)	Maintenance	Response task	RSVP	Relevant	HC	None	0.955	0.001	S
Sambal et al. (2021)	Maintenance	Response task	FL	Relevant	HC	Non‐food	0.561	0.094	G
Donofry et al. (2019)	Orienting	Response task	VS	Irrelevant	HCHC	Non‐food	−0.352	0.227	S
Chen et al. (2023)	Maintenance	Free‐viewing	N/A		HC	LC	−0.304	0.229	S
Hummel et al. (2018)	Maintenance	Free‐viewing	N/A		HC	LC	−1.127	0.001	S
Xu et al. (2023)	Maintenance	Free‐viewing	N/A		HC	Non‐food	0.100	0.468	S
Graham et al. (2011)	Orienting	Free‐viewing	N/A		HC	LC	−1.654	0.022	S
Ahern et al. (2010)	Maintenance	Response task	DP	Irrelevant	Food	Non‐food	−0.279	0.277	S
Brignell et al. (2009)	Maintenance	Response task	DP	Irrelevant	Food	Non‐food	0.178	0.524	U
Hardman et al. (2013)	Maintenance	Response task	DP	Irrelevant	Food	Non‐food	0.455	0.090	U
Kim et al. (2014)	Maintenance	Response task	DP	Irrelevant	Food	Non‐food	0.157	0.656	G
Husted et al. (2016)	Maintenance	Response task	FL	Relevant	Food	Non‐food	0.313	0.297	S
Kirsten et al. (2019)	Orienting	Response task	RSVP	Relevant	Food	Non‐food	−0.515	0.010	S
van Ens et al. (2019)	Maintenance	Instructed viewing	DP	Irrelevant	Food	Non‐food	0.071	0.799	S

Abbreviations: AB, attentional bias; AU, Australia; BMI, body mass index; CA, Canada; CF, chase food; CH, Switzerland; CN, China; Col, college; DE, Germany; DEBQ, Dutch Eating Behavior Questionnaire; DP, dot probe; ED, eating disorder; EDEQ, Eating Disorder Examination Questionnaire; EDU, educational attainment; FL, flanker; G, good; HC, high‐calorie; HE, healthy; IL, Israel; KR, Korea; LC, low‐calorie; NE, neutral; NL, Netherlands; NR, not reported; *p*‐value, significance value; RE, restrained; ROB, risk of bias; RS, restraint scale; RSVP, rapid serial visual presentation; S, satisfactory; SC, spatial cueing; TFEQ, Three Factor Eating Questionnaire; U, unsatisfactory; UK, United Kingdom; Uni, university; URE, unrestrained; USA, United States of America; VS, visual search.

### 3.1 Primary Analysis: Maintenance

#### 3.1.1 General Associations Between Dietary Restraint and Attentional Maintenance

ROB did not influence associations between dietary restraint and maintenance (*B* = 0.084 [−0.271 0.439], *Q* = 0.21, *p* = 0.644; *K* = 25), so all studies were kept in our meta‐analysis. This revealed no significant associations between restraint and attentional maintenance (HG = 0.100 [−0.054, 0.254], *p* = 0.201; Heterogeneity: *Q* = 47.65, *p* = 0.003, *I* ^2^ = 49.6%)—see Supporting Information for forest plot (Figure S1).

#### 3.1.2 Mode of AB Investigation

##### 3.1.2.1 Eye‐Tracking Versus Response Tasks

**FIGURE 2:**
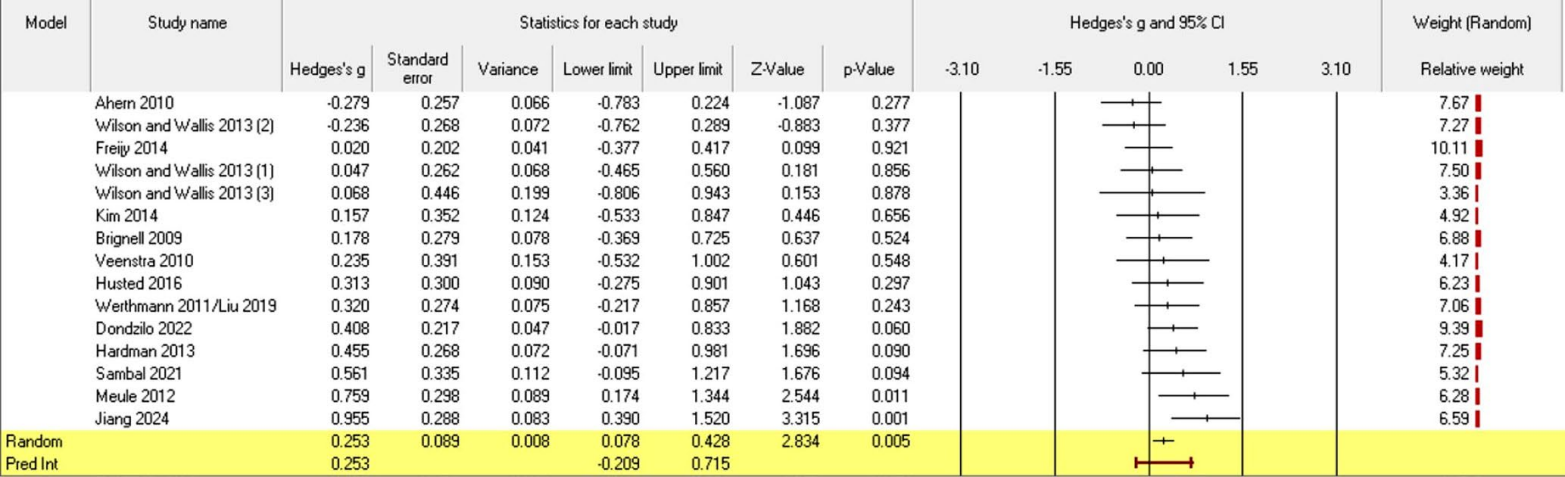
Meta‐analysis of associations between attentional maintenance and dietary restraint in studies using response tasks (*K* = 15).

We compared all response tasks to all eye‐tracking paradigms (i.e., instructed and free‐viewing) and found that response tasks demonstrated positive associations between restraint and attentional maintenance, shown in Figure 2 (HG = 0.253 [0.078, 0.428], *p* = 0.005), but eye‐tracking paradigms did not (HG = −0.129 [−0.370, 0.112], *p* = 0.295; *K* = 10; Figure S2). Furthermore, the comparison test for associations between restraint and maintenance across each group was significant (*Q* = 6.306, *p* = 0.012), and heterogeneity was low‐moderate for response task studies (*Q* = 20.714, *p* = 0.109, *I* ^2^ = 32.4%) and moderate for eye‐tracking paradigms (*Q* = 18.262, *p* = 0.032, *I* ^2^ = 50.7%).

In the unadjusted meta‐regression, the mode of AB investigation coefficient was significant (*B* = 0.377 [0.087, 0.668], *R* ^2^ = 0.28) and the model explained a significant amount of the variance (*Q* = 6.47, *p* = 0.011). Heterogeneity reduced from 49.6% (null model) to 41.0%. Adjusting for clinical ED screening and type of restraint scale improved the precision of the coefficient and heterogeneity (*B* = 0.494 [0.195, 0.793], *p* = 0.001; *R* ^2^ = 0.51; *Q* = 13.36, *p* = 0.010, *I* ^2^ = 31.9%).

Overall, response tasks elicited stronger associations between eating restraint and attentional maintenance compared to all eye‐tracking paradigms, with stronger effects when the variance from type of restraint scale and clinical ED screening were accounted for.

##### 3.1.2.2 Instructed Viewing Versus Free‐Viewing

We sub‐grouped eye‐tracking studies into instructed viewing and free‐viewing to explore differences between all three experimental paradigms. We found no significant associations between restraint and attentional maintenance among free‐viewing studies (HG = −0.162 [−0.733, 0.409], *p* = 0.578; *Q* = 16.733, *p* = 0.001, *I* ^2^ = 82.1%; *K* = 4; Figure S3) nor among instructed viewing studies (HG = −0.116 [−0.352, 0.120], *p* = 0.544; *Q* = 1.308, *p* = 0.934, *I* ^2^ = 0%; *K* = 6; Figure S4). Given the high heterogeneity in the free‐viewing paradigms, we did not run any further analyses using this subgroup.

##### 3.1.2.3 Instructed Viewing Versus Response Tasks

As we previously found a significant association between dietary restraint and ABs in the response task groups, we compared effect sizes between the response task subgroup and instructed viewing subgroup. This comparison showed a significant difference (*Q* = 6.046, *p* = 0.014). In the unadjusted meta‐regression, the mode of AB investigation coefficient was significant (*B* = 0.368 [0.069, 0.667], *R* ^2^ = 0.63) and the model explained a significant amount of the variance (*Q* = 5.82, *p* = 0.016). Heterogeneity reduced from 30.0% (null model) to 13.7%.

Overall, no associations between restraint and attentional maintenance were found for instructed viewing or free‐viewing paradigms. However, when comparing instructed viewing to response tasks, response tasks elicited stronger associations between eating restraint and attentional maintenance.

#### 3.1.3 Response Task Type

**FIGURE 3:**
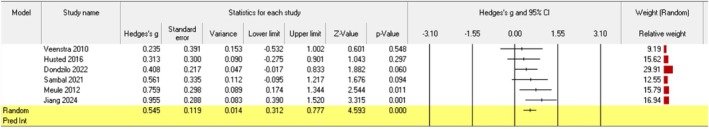
Meta‐analysis of associations between attentional maintenance and dietary restraint in studies using alternative tasks to the dot probe task (*K* = 6).

We explored whether the response task type could account for differential associations between restraint and attentional maintenance. We found no associations between restraint and attentional maintenance among dot probe studies (HG = 0.001 [−0.141, 0.143], *p* = 0.991; *K* = 15; Figure S5) but significant associations among the other tasks group, shown in Figure 3 (HG = 0.545 [0.312, 0.777], *p* = 0.000), with the difference between these effect sizes also significant (*Q* = 15.301, *p* = 0.000). Heterogeneity was low in both the dot probe (*Q* = 9.105, *p* = 0.824, *I* ^2^ = 0%) and alternative tasks group (*Q* = 4.168, *p* = 0.526, *I* ^2^ = 0%).

In the unadjusted meta‐regression, the task type coefficient was significant (*B* = 0.544 [0.271, 0.816]; *R* ^2^ = 1.00) and the model explained a significant amount of the variance (*Q* = 15.30, *p* = 0.000). Heterogeneity was 0% (30.0% in the null model).

Collectively, the alternative tasks were able to capture significant positive associations between restraint and attentional maintenance compared to the dot probe task.

#### 3.1.4 Food Stimuli Relevance

**FIGURE 4:**
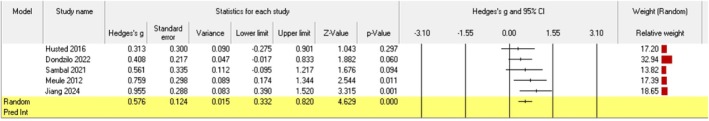
Meta‐analysis of associations between attentional maintenance and dietary restraint in studies using relevant food stimuli (*K* = 5).

We investigated whether the relevancy of food stimuli to the response task could influence associations between restraint and attentional maintenance. The irrelevant stimuli group included the same studies as in the dot probe subgroup, with the addition of one study (Veenstra et al. 2010). The relevant stimuli group included the same studies as the other tasks subgroup minus Veenstra et al. (2010). We found no associations between restraint and attentional maintenance among irrelevant studies (HG = 0.009 [−0.131, 0.148], *p* = 0.904; *K* = 16, Figure S6) and significant associations among the relevant group, shown in Figure 4 (HG = 0.576 [0.332, 0.820], *p* = 0.000), with the differences between these effect sizes also significant (*Q* = 15.644, *p* = 0.000). Heterogeneity was low in the irrelevant group (*Q* = 9.452, *p* = 0.853, *I* ^2^ = 0%) and low in the relevant group (*Q* = 3.478, *p* = 0.481, *I* ^2^ = 0%).

In the unadjusted meta‐regression, the stimuli relevancy coefficient was significant (*B* = 0.567 [0.286, 0.848]; *R* ^2^ = 1.00) and the model explained a significant amount of the variance (*Q* = 15.64, *p* = 0.000). Heterogeneity was 0% (30.0% in the null model).

Overall, task‐relevant food stimuli elicited stronger associations between restraint and attentional maintenance compared to irrelevant food stimuli.

#### 3.1.5 Type of Food Stimuli

We investigated whether the type of food stimuli could influence associations between restraint and attentional maintenance. We found no significant associations among HC studies (0.086 [−0.107, 0.278], *p* = 0.382; *K* = 19; Figure S7) nor among mixed food studies (HG = 0.136 [−0.092, 0.364], *p* = 0.243; *K* = 6; Figure S8). The difference between these effect sizes was also non‐significant (*Q* = 0.108, *p* = 0.743). Heterogeneity was moderate–high in HC group (*Q* = 43.087, *p* = 0.001, *I* ^2^ = 58.2%) and low in the mixed food group (*Q* = 4.455, *p* = 0.486, *I* ^2^ = 0%).

The stimuli type coefficient was non‐significant in the unadjusted meta‐regression (*B* = −0.056 [−0.424, 0.312], *R* ^2^ = −0.09; *Q* = 0.09, *p* = 0.766, *I* ^2^ = 51.6%; *I* ^2^ = 49.6% in null model). We adjusted for clinical ED screening only, given the smaller study number in the mixed food group, with no change in significance (*B* = −0.032 [−0.148, 0.512], *p* = 0.865, *R* ^2^ = −0.10; *Q* = 1.26, *p* = 0.532, *I* ^2^ = 51.8%).

### 3.2 Exploratory Analyses

#### 3.2.1 General Associations Between Dietary Restraint and Attentional Orienting

**FIGURE 5:**
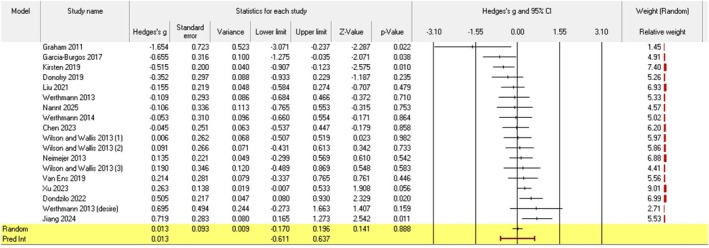
Meta‐analysis of associations between dietary restraint and attentional orienting (*K* = 18).

We assessed ROB using a meta‐regression model and included ROB as the independent variable. ROB was not associated with dataset (*B* = 0.058 [−0.421, 0.536], *Q* = 0.06, *p* = 0.813) so we included all studies in analyses (*K* = 18). The meta‐analysis of the whole dataset revealed no significant associations between restraint and orienting, shown in Figure 5 (HG = 0.013 [−0.170, 0.196], *p* = 0.888; *Q* = 36.754, *p* = 0.004, *I* ^2^ = 53.75%). Heterogeneity was moderate.

Furthermore, we found no significant associations between dietary restraint and orienting in any subgroups of mode of AB investigation, type of response task or stimuli task relevance. Nor did we find any relative differences in restraint‐orienting associations across these subgroups. The mixed food subgroup for type of stimuli only had two samples so we were unable to complete the subgroup analysis and meta‐regression. The HC subgroup showed no significant associations between restraint and orienting (please see Supporting Information for effect sizes and forest plots, Figures S9–S17).

#### 3.2.2 ABs and ED Symptoms

We ran an exploratory analysis to investigate whether ED symptoms may be associated with ABs in the 10 samples that reported or provided data to calculate these associations. Included studies investigated attentional maintenance, mostly captured by the dot‐probe task, and two studies used free‐viewing paradigms (Garcia‐Burgos et al. 2017; Xu et al. 2023), shown in Table 2.

**TABLE 2:** Studies included in ED symptoms analysis (*K* = 10).

Study and paradigm			ED scores and method of analysis				Standardized ES and significance value	
Study	AB mechanism	Experimental paradigm	ED outcome measure	Mean score	Score range	Analysis	Hedge's *G*	*p*
Garcia‐Burgos et al. (2017)	Maintenance	FV	EDE‐Q	1.54	0.06–4.38	Correlation	0.345	0.257
van Ens et al. (2019)	Maintenance	DP	EDE‐Q (SHORT)	2.53	0–10	Correlation	0.901	0.003
Kim et al. (2014)	Maintenance	DP	EDE‐Q KOREAN VERSION	5.65	0.00–15.85	Correlation	−0.063	0.859
Ahern et al. (2010)	Maintenance	DP	EDDS	17.5	Missing	Correlation	−0.599	0.025
Wilson and Wallis (2013) (1)	Maintenance	DP	EDI‐2	13.59	Missing	Correlation	−0.142	0.587
Wilson and Wallis (2013) (2)	Maintenance	DP	EDI‐2	13.26	Missing	Correlation	−0.571	0.039
Wilson and Wallis (2013) (3)	Maintenance	DP	EDI‐2	15.53	Missing	Correlation	0.076	0.825
Brignell et al. (2009)	Maintenance	DP	EAT	10.23	Missing	Correlation	0.299	0.289
Xu et al. (2023)	Maintenance	FV	TSF	31.74	14–65	Correlation	0.302	0.029
Nannt et al. (2025)	Maintenance	DP	EDE‐Q	0.26	Missing	Correlation	−0.158	0.639

Abbreviations: AB, attentional bias; DP, dot‐probe; EAT, Eating Attitudes Scale; ED, eating disorder; EDDS, Eating Disorder Diagnostic Scale; EDE‐Q, Eating Disorder Examination Questionnaire; EDI‐2, Eating Disorder Inventory; FV, free‐viewing; TSF, Thought Shape Fusion Questionnaire.

**FIGURE 6:**
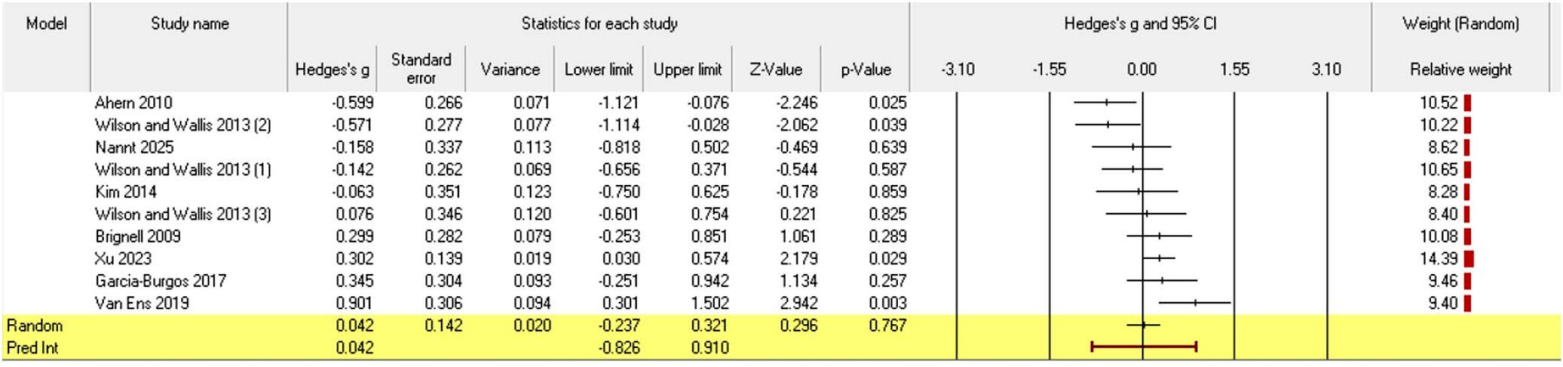
Meta‐analysis of associations between ED symptoms and attentional maintenance (*K* = 10).

We found no associations between ABs and ED symptoms: HG = 0.042 [−0.237, 0.321], *p* = 0.767; *Q* = 24.499, *p* = 0.004, *I* ^2^ = 63.26%, shown in Figure 6. Heterogeneity was moderate‐high among studies. We explored whether controlling for whether clinical EDs were screened out could reveal an association between attentional maintenance and ED symptoms. The overall model and coefficient remained non‐significant (*B* = 0.025 [−0.613, 0.662], *p* = 0.939, *R* ^2^ = −0.28; *Q* = 0.01, *p* = 0.939, *I* ^2^ = 66.8%).

We also explored whether associations between ABs and ED symptoms may differ across ED outcome measures. We created three subgroups so that each group had a minimum of three samples to analyze: EDEQ (*K* = 4), *EDI‐2* (*K* = 3), *Other* (*K* = 3). No significant associations were found between ABs and ED symptoms in each subgroup: *EDEQ* HG = 0.275 [−0.203, 0.752], *p* = 0.259, *Q* = 6.792, *p* = 0.079, *I* ^2^ = 55.83%; *EDI‐2* HG = −0.243 [−0.603, 0.118], *p* = 0.187, *Q* = 2.402, *p* = 0.301, *I* ^2^ = 16.73%; *K* = 3; *Other* HG = 0.022 [−0.531, 0.575], *p* = 0.939, *Q* = 9.369, *p* = 0.009, *I* ^2^ = 78.65% (Please see Figures S18–S20).

### 3.3 Publication Bias

Although some subgroups had less than 10 studies (Page, McKenzie, et al. 2021; Page, Sterne, et al. 2021), we still checked for publication bias in each of our subgroups using Egger's Test and visual inspections of funnel plots. We found no evidence of publication bias (please see Supporting Information for funnel plots and Egger's Test *p*‐values, Figures S21–S30).

## 4 Discussion

Existing evidence indicates that dietary restraint may contribute to the development and maintenance of EDs, with food‐related ABs as a key underlying mechanism. So far, only one study provided a systematic synthesis of the literature on ABs and dietary restraint, which was largely inconclusive due to high statistical heterogeneity between studies (Watson and Le Pelley 2021). To reduce such heterogeneity, our work focused on one cognitive bias—that is, ABs—and isolated its different mechanisms by distinguishing between orienting and maintenance processes. In addition, we conducted analyses to assess how various factors, including the mode of AB investigation, the type of response task, the relevance or irrelevance of stimuli to the task, and the characteristics of food‐related stimuli, may influence the link between ABs and dietary restraint.

When investigating the type of AB mechanism across each dataset, we found no significant associations between restraint and attentional maintenance or attentional orienting. These results were unsurprising, as we hypothesized that different methodological features could contribute to the inconsistent findings observed in previous studies. This aligns with the conclusions of Watson and Le Pelley (2021), who suggested that unaccounted heterogeneity could be masking these associations.

When investigating the influence of our chosen methodological factors, we only found significant associations between dietary restraint and ABs for attentional maintenance indices, but not for orienting indices. The absence of preferential orienting to food‐related cues among dietary restrainers suggests a lack of a bottom‐up, stimulus‐driven processing of food cues. This contrasts with findings in those with clinical EDs, who consistently demonstrate food‐related orienting ABs (Meregalli et al. 2023; Schmitz et al. 2014). Such orienting biases have been interpreted as evidence of reward (Schag et al. 2013) or threat‐driven vigilance (Booth 2014; Radix et al. 2023), which may either encourage binge eating (Schag et al. 2013) or drive severe energy restriction (Meregalli et al. 2023). Although this pattern might initially appear inconsistent with the conceptualization of dietary restraint as a risk factor for EDs, it does align with some evidence suggesting that subclinical disordered eating may be more strongly associated with attentional maintenance (Jin et al. 2023; Soleymani et al. 2022) than orienting (Freccero et al. 2025), possibly reflecting better self‐regulatory control over food cues in those below a clinical threshold for EDs. As a result, ABs in ED‐vulnerable groups, such as dietary restrainers, may still predominantly manifest as maintenance rather than orienting, reflecting purposeful, top‐down processing (Grubb et al. 2015) and a more goal‐directed attentional strategy.

Furthermore, we found significant associations between dietary restraint and attentional maintenance when stimuli were relevant, rather than irrelevant, to the task. This corroborates the idea that dietary restrainers activate self‐regulatory processes when food is relevant to their current goals and actions (Papies and Hamstra 2010).

Taken together, our findings suggest that such top‐down processing in food‐relevant contexts could indicate more strategic monitoring that facilitates dietary restraint (Johnson et al. 2012; Moore et al. 2022). The degree of success in dietary restraint may indeed be influential in our findings. While successful restrainers (i.e., maintaining restraint without periods of disinhibited eating; Johnson et al. 2012) may bypass bottom‐up, stimulus driven attention (i.e., orienting), in favor of more purposeful monitoring in relevant contexts, unsuccessful restrainers, who tend to show higher ED pathology and maladaptive regulation of eating behaviors (Meule, Papies, and Kübler 2012; Meule, Vögele, and Kübler 2012), may manifest both more orienting (Jiang et al. 2024; Alblas et al. 2020) and maintenance (Weng et al. 2012) regardless of context. In line with this reasoning, we found no associations between dietary restraint and attentional maintenance across HC or mixed food stimuli, nor any relative group differences. This could reflect strategic downregulation of the motivational salience system (Xie and Chen 2025), reducing ABs to diet‐incongruent cues (Weng et al. 2012; Higgs et al. 2015). In turn, this may facilitate successful, rather than unsuccessful restraint, whereby heightened motivational salience to HC cues can override energy homeostasis (Xie and Chen 2025) and reinforce the counterregulatory eating (Hagerman et al. 2021) that contributes to binge‐eating pathology (Schag et al. 2013).

We tried to confirm whether the degree of success in dietary restraint influenced our findings; however, not enough papers made this distinction. Instead, we ran a meta‐analysis of correlations between ED symptoms and ABs in the hope to indirectly gauge maladaptive forms of dietary restraint. However, we found no associations between maintenance and global ED symptoms. It is possible that a threshold effect may exist, whereby associations are more likely to emerge when ED symptoms reach a certain level of severity (Soleymani et al. 2022), perhaps not captured if more successful restrainers were included in our samples (Meule, Papies, and Kübler 2012; Meule, Vögele, and Kübler 2012). Global ED symptom scores may have also masked more specific associations between attentional maintenance and dietary restraint, which may have emerged if considering specific ED profiles such as binge‐eating (Sablottny et al. 2025) or restrictive symptoms (Mercado et al. 2020; Meregalli et al. 2023). In addition, as attentional orienting is linked to disorder‐driven saliency processing across different EDs (Meregalli et al. 2023; Schmitz et al. 2014; Sablottny et al. 2025), this AB mechanism may instead show stronger associations with global ED pathology.

The body of literature on dietary restraint and ABs also diverges in the way in which ABs are investigated, which may account for some of the conflicting findings. Specifically, we investigated AB investigation mode and type of response task. We found significant dietary restraint and maintenance associations in the response tasks group versus the eye‐tracking group. One possible explanation lies in the increased AB index consistency in the response tasks compared to the eye‐tracking indices. For instance, gaze fixations have no standardized definition (e.g., Hummel et al. 2018; Liu et al. 2021; Chen et al. 2023). Another source of variance was the inclusion of both free‐viewing and instructed‐viewing paradigms, with free‐viewing being too heterogeneous to glean any meaningful differences between them.

We also found significant associations between dietary restraint and maintenance in the alternative response tasks compared to the dot‐probe. While this may at first be surprising, as the alternative tasks group was inevitably a less homogenous group, it is in line with what is consistently reported in the literature regarding the unreliability of the dot probe task as a measure of AB (Price et al. 2015; Chapman et al. 2019; Xu et al. 2025). Moreover, in line with the significant effects found for task‐relevant food stimuli, we note that five out of six alternative‐task studies used stimuli that were relevant to the task instructions. Therefore, this finding may be attributable to the relevance of the food stimuli to the task, reflecting top‐down attentional processing in dietary restrainers.

Our results need to be considered in light of some limitations. First, we only considered a select number of methodological factors, and other existing factors, such as the induction of certain moods (Hepworth et al. 2010) or homeostatic states (Feighan et al. 2025) could also influence associations between dietary restraint and ABs. Second, despite our efforts, we were unable to retrieve data from 24 studies, which made our results somewhat incomplete. Third, due to the constraints imposed by univariate analytical methods, we were forced to prioritize specific factors, leading to the exclusion of specific stimuli comparisons, modes of AB investigation, attentional mechanisms and gaze and performance indices. Lastly, our investigations of attentional orienting and ED symptoms were exploratory only and should be confirmed in future research. Although the following points are not directly related to this meta‐analysis, they represent shortcomings in the literature that warrant consideration. First, most of our samples were young adult females from universities in Europe and the UK, and we were unable to make further inferences about race or ethnicity as only three studies reported this information. Furthermore, as only two studies reported socioeconomic information, we could not explore the potential impact of any socioeconomic variables. Hence, the generalizability of our findings remains unclear. Second, while we made efforts to screen out studies involving clinical samples exclusively, some studies did not screen for clinical EDs so may have captured individuals above the clinical threshold. Where possible, we controlled for whether clinical ED screening took place, to extend findings to non‐clinical groups who may therefore be at risk of ED development. As data on dietary restraint success level was not available, it remains unknown whether, unsuccessful dietary restrainers (i.e., a group that may be at higher risk of ED development) may show stronger associations between ABs and ED symptoms than successful restrainers.

Overall, our meta‐analysis highlights that some of the inconsistencies observed in the relationship between dietary restraint and ABs could be attributable to varying methodological approaches used in the literature. Specifically, our findings suggest that dietary restrainers may show strategic top‐down processing of food cues when food is relevant to their goals and actions, and certain response tasks may be more reliable in capturing this phenomenon. Furthermore, attentional orienting may not reliably characterize food cue processing in dietary restrainers. Future research should explore how dietary restraint may relate to differing AB mechanisms, and whether the degree of restraint success is influential in this. Further research is also needed to confirm whether such associations are related to ED pathology. This should be explored in populations that tend to be underrepresented in ED research, such as males and minority ethnic groups. This would help clarify whether unsuccessful restraint is more strongly associated with AB mechanisms that indicate maladaptive salience processing, and whether this contributes to a higher risk of ED development across populations.

## Author Contributions

**Rio Madan:** conceptualization, methodology, formal analysis, data curation, writing – original draft, writing – review and editing. **Cristina Martinelli:** supervision, conceptualization, methodology, formal analysis, writing – original draft, writing – review and editing.

## Funding

The authors have nothing to report.

## Lived Experience Involvement Statement

No specific efforts were undertaken to involve persons with lived experience in the study design or execution, or in the preparation of this manuscript.

## Conflicts of Interest

The authors declare no conflicts of interest.

## Supporting information


**Data S1:** ROB criteria explained.

**Data S2:** ROB table of final scores.

**Figure S1:** Meta‐analysis of associations between attentional maintenance and dietary restraint (*K* = 25).
**Figure S2:** Meta‐analysis of associations between attentional maintenance and dietary restraint in studies using eye‐tracking (*K* = 10).
**Figure S3:** Meta‐analysis of associations between attentional maintenance and dietary restraint in studies using free‐viewing (*K* = 4).
**Figure S4:** Meta‐analysis of associations between attentional maintenance and dietary restraint in studies using instructed‐viewing (*K* = 6).
**Figure S5:** Meta‐analysis of associations between attentional maintenance and dietary restraint in studies using the dot probe task (*K* = 15).
**Figure S6:** Meta‐analysis of associations between attentional maintenance and dietary restraint in studies using irrelevant food stimuli (*K* = 16).
**Figure S7:** Meta‐analysis of associations between attentional maintenance and dietary restraint in studies using HC stimuli (*K* = 19).
**Figure S8:** Meta‐analysis of associations between attentional maintenance and dietary restraint in studies using mixed food stimuli (*K* = 6).
**Figure S9:** Meta‐analysis of associations between restraint and orienting using response tasks (*K* = 8).
**Figure S10:** Meta‐analysis of associations between restraint and orienting using eye‐tracking (*k* = 10).
**Figure S11:** Meta‐analysis of associations between restraint and orienting using free‐viewing (*K* = 4).
**Figure S12:** Meta‐analysis of associations between restraint and orienting using instructed‐viewing (*k* = 6).
**Figure S13:** Meta‐analysis of associations between restraint and orienting using dot probe task (*k* = 9).
**Figure S14:** Meta‐analysis of associations between restraint and orienting using alternative tasks (*K* = 5).
**Figure S15:** Meta‐analysis of associations between restraint and orienting using irrelevant stimuli (*k* = 10).
**Figure S16:** Meta‐analysis of associations between restraint and orienting relevant stimuli (*k* = 4).
**Figure S17:** Meta‐analysis of associations between restraint and orienting using HC stimuli (*K* = 16).
**Figure S18:** Meta‐analysis of associations between maintenance and ED symptoms using the EDEQ (*K* = 4).
**Figure S19:** Meta‐analysis of associations between maintenance and ED symptoms using the EDI‐2 (*K* = 3).
**Figure S20:** Meta‐analysis of associations between maintenance and ED symptoms using different outcome measures (*K* = 3).
**Figure S21:** Funnel plot of all included samples, *p* = 0.967.
**Figure S22:** Funnel plot of Response Tasks, *p* = 0.507.
**Figure S23:** Funnel plot of Instructed‐viewing paradigms, *p* = 0.320.
**Figure S24:** Funnel plot of Free‐viewing paradigms, *p* = 0.324.
**Figure S25:** Funnel plot of Dot probe studies, *p* = 0.918.
**Figure S26:** Funnel plot of alternative task studies, *p* = 0.999.
**Figure S27:** Funnel plot of relevant stimuli, *p* = 0.546.
**Figure S28:** Funnel plot of Irrelevant stimuli, *p* = 0.882.
**Figure S29:** Funnel plot of HC stimuli, *p* = 0.952.
**Figure S30:** Funnel plot of Mixed food stimuli, *p* = 0.573.

## Data Availability

Data and materials will be made available on request by the corresponding author.
