# Supplementary material for: Food‐Related Attentional Biases in Restrained Eaters: A Meta‐Analysis
Source: Int J Eat Disord. 2026 Mar 31;59(7):1426–44. doi: 10.1002/eat.70090 (PMC13326802; doi:10.1002/eat.70090)
Supplement: Supplementary file 1 — Data S1: ROB criteria explained. [file EAT-59-1426-s002.docx]

Newcastle Ottawa Scale (NOS) for Risk of Bias – adapted for cross-sectional studies

Selection: (maximum 5 stars)

1. **Representativeness of the sample (1 star total):**
   1. Truly representative of the target population. *
   2. Somewhat representative of the target group. *
   3. Selected group of users/convenience sample.
   4. No description of the derivation of the included subjects.

**A** – When studies recruited in the community (any group) or in two or more universities, as this would have captured a wider variety of students/staff versus just one university.

**B** – When studies recruited within just one university. We included samples described as “students”, even if the particular recruitment methods were not described.

**C** – When studies recruited within a particular class of a university, e.g. first year psychology students.

**D** – When no description of where the sample was recruited from.

1. **Are any conditions that can affect eating behaviours screened out? Chosen items are as follows: 1) any eating disorder, 2) any medical condition that affects eating (e.g. allergies). Items are counted as “one” even if more than one condition from the same group is screened out. (2 stars total)**
2. 1 or more items are screened out by validated measure **
3. 1 or more items are screened out by self-report*
4. No items screened out

A validated measure referred to any standardised outcome measure to assess behaviours or conditions. Self-report included non-specific questionnaires, or no description of the specific tool used for screening.

1. **Was restraint status assessed with validated measure regardless of study design? (1 star total)**

a. Restraint status assessed with validated measure*

c. Restraint status asked to individuals

d. No description

A validated measure referred to any standardised outcome measure to assess eating restraint.

1. **Sample size (1 star total):**
   1. Justified and satisfactory (including sample size calculation). *
   2. Not justified.
   3. No information provided

To gain a point, we included a priori calculations, post hoc power analyses and justifications using previous similar research if minimum sample size was unknown.

Comparability: (Maximum 6 stars)

1. **The subjects in different groups are comparable. If not a between group design, then confounding/predictive factors are controlled for – these would be age, sex, education, IQ, Working Memory, BMI, hunger, appetite, time since last meal, food craving, stimuli palatability ratings (2 stars total)**
   1. Groups matched for at least 2 from the list above OR at least 2 factors controlled for in the analyses**
   2. Groups matched for at least 1 from the list above OR at least 1 factor controlled for in the analyses*
   3. Factors not included in the list above
   4. None

Between-groups analyses (e.g. high versus low restraint) must have reported the statistical test to demonstrate matched variables between restraint groups. Points were also allocated if potential confounders were included as covariates in ANOVA models (ANCOVA) should they have differed between groups. Correlational analyses between restraint and ABs must have included variables as a control factor in the statistical model.

For both between-groups and correlations, we did not allocate points for assessing associations between AB outcomes and potential confounders (separately from the main analysis), even if no associations were found. This is because potential confounders may still impact the overall precision of results. We also wanted to stay aligned with previous literature that reports associations between ABs and our chosen confounders.

We allocated points for “time since last meal” if participants were told to fast or eat at a certain time before attending the session, regardless of whether adherence was checked by researchers.

We allocated points when raw data was received to carry out analyses between restraint and AB outcomes, and these data included potential confounding variables that could be controlled. We also allocated points if we received only the outcome data from authors and confounding variables were reported to be controlled.

1. **Additional criteria for paradigm conditions (4 stars total)**

- Restraint is administered before task to control for exposure to food tasting/images impacting on restraint scores OR significant time period after testing session*
- Standardised database used for stimuli and stated visual property matching completed by database or by objective measures (e.g. software used)*
- Recognisability of stimuli groups checked and findings reported*
- Palatability of stimuli groups checked and findings reported*

For this question, studies gained one point per fulfilled criterion.

**Database Point**

We allocated points when one or more standardised databases were reported that included information on the visual properties of stimuli. These standardised databases did not require an additional objective measure to match visual properties to gain a point.

We allocated points if non-standardised sources of stimuli were reported but any form of software or objective measure was used to match the stimuli.

We did not allocate points when no standardised database was reported along with no objective matching methods. Non-standardised sources included various internet sources, photographs, references to stimuli of previous studies that were also non-standardised, descriptions of stimuli being “similar to” those of previous studies even if from a standardised source.

Visual properties included (but were not limited to) brightness, background colour or size.

**Recognisability and Palatability Points**

Each of these points were allocated whether the current sample or a different sample were used.

Recognisability referred to ensuring participants categorised each picture as the study’s intended category, e.g. a high calorie stimulus is correctly categorised as high calorie and not low calorie. We allocated points as long as authors reported they had carried this out, regardless of the actual results.

Palatability may have also been described as *liking, arousal, valence, tastiness, enjoyment* or any other adjective describing the tastiness of the stimuli for participants. Again, we allocated points as long as authors reported they had carried this out, regardless of the actual results. We included this variable in the comparability section to award points if it was controlled for.

Outcome: (max 3 stars)

1. **Trials and randomisation (2 stars total):**

- Randomisation is included
- At least one method of reducing fatigue effects is reported (e.g. random ITI, breaks between blocks/trials, filler trials)
- Transformation of AB index is clearly stated
- Trial data exclusions and outliers fully described

** = Randomisation + 2

* = Randomisation + 1

For this question, randomisation plus (at least) one criterion had to be included to gain at least one point.

**Randomisation**

Randomisation referred to the order in which food stimuli were shown in each trial. We did not allocate points if randomisation was used for anything other than the stimulus itself, e.g. the position of stimuli on the screen.

**Fatigue Effects**

ITI (inter-trial-interval) referred to the time in between each trial in the task. We allocated points if any variation was used, e.g. completely random or distinct patterns of change.

Breaks in between blocks or trials had to be described as breaks and distinguished from the ITI in order to gain a point.

Filler trials are not typically included in analyses and are usually of stimuli unrelated to the study topic. No restrictions were placed on filler trials, as long as they were described as such, or indicated that their inclusion was to aid participant concentration/maintain unpredictability.

**AB Index**

The AB index referred to the chosen method of calculating an AB from the mean performance on the task/eye-tracking indices. This included subtracting mean reaction time for non-food stimuli from mean reaction time for food stimuli (RTfood – RTnonfood) to indicate a food specific AB. We allocated points for any method used, as long as it was clearly described.

**Trial Data Exclusions**

This referred to the process of cleaning the trial data. This mainly included information about outliers and incorrect trials, but we also allocated points for other relevant information on preparing the trial data for analyses. We did not allocate points if no information on this was provided.

1. **Statistical test (1 star total):**
   1. Statistical test used to analyse the data clearly described, appropriate and measures of association presented and probability level (p value). *
   2. Statistical test not appropriate, not described or incomplete.

We did not allocate points if studies did not report key descriptive statistics of outcome measures used and/or key descriptive statistics of main AB task performance.

We did not allocate points if associations between ABs and restraint were a key focus of the study, but results (even if non-significant) were not fully reported (or available in supplementary material), rendering data incomplete for our meta-analyses. However, if we received the needed data upon request, points were then allocated.

**Total out of 14**

- Very Good = 13-14
- Good = 10-12
- Satisfactory = 7-9
- Unsatisfactory = 0-6

Scores were rescaled from the version of the NOS (Wells et al, 2000) that we adapted (Herzog et al, 2013). They were rescaled from a maximum of 10 points to 14 points using the linear rescaling method.

**References**

Herzog, R., Álvarez-Pasquin, M. J., Díaz, C., Del Barrio, J. L., Estrada, J. M., & Gil, Á. (2013). Are healthcare workers’ intentions to vaccinate related to their knowledge, beliefs and attitudes? A systematic review. *BMC public health*, *13*, 1-17.

Wells, G. A., Shea, B., O’Connell, D., Peterson, J., Welch, V., Losos, M., & Tugwell, P. (2000). The Newcastle-Ottawa Scale (NOS) for assessing the quality of nonrandomised studies in meta-analyses.
