# Supplementary material for: Food‐Related Attentional Biases in Restrained Eaters: A Meta‐Analysis
Source: Int J Eat Disord. 2026 Mar 31;59(7):1426–44. doi: 10.1002/eat.70090 (PMC13326802; doi:10.1002/eat.70090)
Supplement: Supplementary file 2 — Data S2: ROB table of final scores. [file EAT-59-1426-s001.docx]

**Risk of Bias Final Scores**

| STUDY | SELECTION (5) | COMPARABILITY (6) | OUTCOME (3) | FINAL SCORE (14) | RISK CATEGORY |
| --- | --- | --- | --- | --- | --- |
| Ahern 2010 | 1 | 4 | 3 | 8 | S |
| Brignell 2009 | 2 | 1 | 3 | 6 | U |
| Chen 2023 | 2 | 6 | 1 | 9 | S |
| Dondzilo 2022 | 2 | 3 | 3 | 8 | S |
| Freijy 2014 | 2 | 2 | 3 | 7 | S |
| Graham 2011 | 3 | 3 | 1 | 7 | S |
| Garcia-Burgos 2017 | 4 | 5 | 3 | 12 | G |
| Hardman 2013 | 2 | 0 | 1 | 3 | U |
| Hummel 2018 | 2 | 4 | 3 | 9 | S |
| Husted 2016 | 3 | 3 | 2 | 8 | S |
| Jiang 2024 | 4 | 3 | 2 | 9 | S |
| Kim 2014 | 3 | 5 | 3 | 11 | G |
| Kirsten 2019 | 3 | 4 | 2 | 9 | S |
| Werthmann 2011/Liu 2019 | 3 | 1 | 3 | 7 | S |
| Werthmann 2013 | 1 | 2 | 3 | 6 | U |
| Werthmann 2013 (desire) | 2 | 1 | 3 | 6 | U |
| Werthmann 2014 | 2 | 1 | 3 | 6 | U |
| Liu 2021 | 3 | 3 | 3 | 9 | S |
| Meule 2012 | 1 | 2 | 1 | 4 | U |
| Neimeijer 2013 | 1 | 3 | 3 | 7 | S |
| Sambal 2021 | 4 | 3 | 3 | 10 | G |
| Van Ens 2019 | 3 | 3 | 3 | 9 | S |
| Veenstra 2010 | 1 | 4 | 2 | 7 | S |
| Wallis 2013 (1) | 3 | 2 | 1 | 6 | U |
| Wallis 2013 (2) | 3 | 3 | 1 | 7 | S |
| Wallis 2013 (3) | 3 | 3 | 1 | 7 | S |
| Xu 2023 | 2 | 6 | 1 | 9 | S |
| Donofry 2019 | 3 | 2 | 3 | 8 | S |
| Nannt 2025 | 5 | 4 | 3 | 12 | G |

*S: satisfactory; G: good; U: unsatisfactory*
