# Supplementary material for: Food‐Related Attentional Biases in Restrained Eaters: A Meta‐Analysis
Source: Int J Eat Disord. 2026 Mar 31;59(7):1426–44. doi: 10.1002/eat.70090 (PMC13326802; doi:10.1002/eat.70090)

**Search Strategy – 11^th^ April 2024, 31^st^ October 2024, 25^th^ July 2025, 17^th^ December, 2025**

**Web of Science**

(Attent* OR bias* OR avoid* OR ignor* OR reject* OR desir* OR attract* OR select* OR prefer* OR engag* OR disengag* OR orient* OR gaz* OR approach* OR distract* OR focus*) AND ((“food* “NEAR/4” image*”) OR (“calori* “NEAR/4” image*”) OR (“nutri* “NEAR/4” image*”) OR (“meal* “NEAR/4” image*”) OR (“food* “NEAR/4” pic*”) OR (“calori* “NEAR/4” pic*”) OR (“nutri* “NEAR/4” pic*”) OR (“meal* “NEAR/4” pic*”) OR (“food* “NEAR/4” stimul*”) OR (“calori* “NEAR/4” stimul*”) OR (“nutri* “NEAR/4” stimul*”) OR (“meal* “NEAR/4” stimul*”) OR “food* info*” OR “calori* info*” OR “nutri* info*” OR “meal* info*” OR “food* content*” OR “calori* content*” OR “nutri* content*” OR “meal* content*” OR “visual cue*” OR “food* cue*”) AND (“diet* restr*” OR “eat* restr*” OR diet* OR nondiet* OR unrestr* OR restr* OR vegan* OR vegetarian* OR “ultra-process*” OR pescatarian* OR “low* carb* diet*” OR “low* fat* diet*” OR “low* sugar* diet*” OR “low* calori* diet*” OR omnivore* OR fast* OR “dairy free”)

**PsycInfo**

TI (Attent* OR bias* OR avoid* OR ignor* OR reject* OR desir* OR attract* OR select* OR prefer* OR engag* OR disengag* OR orient* OR gaz* OR approach* OR distract* OR focus*) OR AB (Attent* OR bias* OR avoid* OR ignor* OR reject* OR desir* OR attract* OR select* OR prefer* OR engag* OR disengag* OR orient* OR gaz* OR approach* OR distract* OR focus*)

AND TI ((“food* N4 image*”) OR (“calori* N4 image*”) OR (“nutri* N4 image*”) OR (“meal* N4 image*”) OR (“food* N4 pic*”) OR (“calori* N4 pic*”) OR (“nutri* N4 pic*”) OR (“meal* N4 pic*”) OR (“food* N4 stimul*”) OR (“calori* N4 stimul*”) OR (“nutri* N4 stimul*”) OR (“meal* N4 stimul*”) OR “food* info*” OR “calori* info*” OR “nutri* info*” OR “meal* info*” OR “food* content*” OR “calori* content*” OR “nutri* content*” OR “meal* content*” OR “visual cue*” OR “food* cue*”) OR AB ((“food* N4 image*”) OR (“calori* N4 image*”) OR (“nutri* N4 image*”) OR (“meal* N4 image*”) OR (“food* N4 pic*”) OR (“calori* N4 pic*”) OR (“nutri* N4 pic*”) OR (“meal* N4 pic*”) OR (“food* N4 stimul*”) OR (“calori* N4 stimul*”) OR (“nutri* N4 stimul*”) OR (“meal* N4 stimul*”) OR “food* info*” OR “calori* info*” OR “nutri* info*” OR “meal* info*” OR “food* content*” OR “calori* content*” OR “nutri* content*” OR “meal* content*” OR “visual cue*” OR “food* cue*”)

AND TI (“diet* restr*” OR “eat* restr*” OR diet* OR nondiet* OR unrestr* OR restr* OR vegan* OR vegetarian* OR “ultra-process*” OR pescatarian* OR “low* carb* diet*” OR “low* fat* diet*” OR “low* sugar* diet*” OR “low* calori* diet*” OR omnivore* OR fast* OR “dairy free”) OR AB (“diet* restr*” OR “eat* restr*” OR diet* OR nondiet* OR unrestr* OR restr* OR vegan* OR vegetarian* OR “ultra-process*” OR pescatarian* OR “low* carb* diet*” OR “low* fat* diet*” OR “low* sugar* diet*” OR “low* calori* diet*” OR omnivore* OR fast* OR “dairy free”)

**MEDLINE**

TI (Attent* OR bias* OR avoid* OR ignor* OR reject* OR desir* OR attract* OR select* OR prefer* OR engag* OR disengag* OR orient* OR gaz* OR approach* OR distract* OR focus*) OR AB (Attent* OR bias* OR avoid* OR ignor* OR reject* OR desir* OR attract* OR select* OR prefer* OR engag* OR disengag* OR orient* OR gaz* OR approach* OR distract* OR focus*)

AND TI ((“food* N4 image*”) OR (“calori* N4 image*”) OR (“nutri* N4 image*”) OR (“meal* N4 image*”) OR (“food* N4 pic*”) OR (“calori* N4 pic*”) OR (“nutri* N4 pic*”) OR (“meal* N4 pic*”) OR (“food* N4 stimul*”) OR (“calori* N4 stimul*”) OR (“nutri* N4 stimul*”) OR (“meal* N4 stimul*”) OR “food* info*” OR “calori* info*” OR “nutri* info*” OR “meal* info*” OR “food* content*” OR “calori* content*” OR “nutri* content*” OR “meal* content*” OR “visual cue*” OR “food* cue*”) OR AB ((“food* N4 image*”) OR (“calori* N4 image*”) OR (“nutri* N4 image*”) OR (“meal* N4 image*”) OR (“food* N4 pic*”) OR (“calori* N4 pic*”) OR (“nutri* N4 pic*”) OR (“meal* N4 pic*”) OR (“food* N4 stimul*”) OR (“calori* N4 stimul*”) OR (“nutri* N4 stimul*”) OR (“meal* N4 stimul*”) OR “food* info*” OR “calori* info*” OR “nutri* info*” OR “meal* info*” OR “food* content*” OR “calori* content*” OR “nutri* content*” OR “meal* content*” OR “visual cue*” OR “food* cue*”)

AND TI (“diet* restr*” OR “eat* restr*” OR diet* OR nondiet* OR unrestr* OR restr* OR vegan* OR vegetarian* OR “ultra-process*” OR pescatarian* OR “low* carb* diet*” OR “low* fat* diet*” OR “low* sugar* diet*” OR “low* calori* diet*” OR omnivore* OR fast* OR “dairy free”) OR AB (“diet* restr*” OR “eat* restr*” OR diet* OR nondiet* OR unrestr* OR restr* OR vegan* OR vegetarian* OR “ultra-process*” OR pescatarian* OR “low* carb* diet*” OR “low* fat* diet*” OR “low* sugar* diet*” OR “low* calori* diet*” OR omnivore* OR fast* OR “dairy free”)

**PubMed**

(“Attent*” OR “bias*” OR “avoid*” OR “ignor*” OR “reject*” OR “desir*” OR “attract*” OR “select*” OR “prefer*” OR “engag*” OR “disengag*” OR “orient*” OR “gaze*” OR “gazing” OR “approach*” OR “distract*” OR “focus*”) AND ((“food image” [tiab:~4]) OR (“food images” [tiab:~4]) OR (“calorie image” [tiab:~4]) OR (“calorie images” [tiab:~4]) OR (“calories images” [tiab:~4]) (“caloric image” [tiab:~4]) OR (“caloric images” [tiab:~4]) OR (“nutrition image” [tiab:~4]) OR (“nutritional image” [tiab:~4]) OR (“nutrition images” [tiab:~4]) OR (“nutritional images” [tiab:~4]) OR (“nutrient image” [tiab:~4]) OR (“nutrient images” [tiab:~4]) OR (“nutrients images” [tiab:~4]) OR (“meal image” [tiab:~4]) OR (“meal images” [tiab:~4]) OR (“meals image” [tiab:~4]) OR (“meals images” [tiab:~4]) OR (“food pic” [tiab:~4]) OR (“food pics” [tiab:~4]) OR (“food picture” [tiab:~4]) OR (“food pictures” [tiab:~4]) OR (“foods pictures” [tiab:~4]) OR (“foods picture” [tiab:~4]) OR (“calorie pic” [tiab:~4]) OR (“calorie pics” [tiab:~4]) OR (“calorie picture” [tiab:~4]) OR (“calorie pictures” [tiab:~4]) OR (“calories pictures” [tiab:~4]) OR (“caloric pic” [tiab:~4]) OR (“caloric pics” [tiab:~4]) OR (“caloric picture” [tiab:~4]) OR (“caloric pictures” [tiab:~4]) OR (“nutrition pic” [tiab:~4]) OR (“nutrition pics” [tiab:~4]) OR (“nutrition pictures” [tiab:~4]) OR (“nutrition picture” [tiab:~4]) OR (“nutrient pic” [tiab:~4]) OR (“nutrient pics” [tiab:~4]) OR (“nutrient picture” [tiab:~4]) OR (“nutrient pictures” [tiab:~4]) OR (“nutrients pictures” [tiab:~4]) OR (“nutritional pictures” [tiab:~4]) OR (“nutritional picture” [tiab:~4]) OR (“nutritional pic” [tiab:~4]) OR (“nutritional pics” [tiab:~4]) OR (“meal pic” [tiab:~4]) OR (“meal pics” [tiab:~4]) OR (“meals pics” [tiab:~4]) OR (“meal picture” [tiab:~4]) OR (“meal pictures” [tiab:~4]) OR (“meals pictures” [tiab:~4]) OR (“food stimuli” [tiab:~4]) OR (“food stimulus” [tiab:~4]) OR (“foods stimuli” [tiab:~4]) OR (“calorie stimuli” [tiab:~4]) OR (“calorie stimulus” [tiab:~4]) OR (“calories stimuli” [tiab:~4]) OR (“caloric stimuli” [tiab:~4]) OR (“caloric stimulus” [tiab:~4]) OR (“nutrition stimuli” [tiab:~4]) OR (“nutrition stimulus” [tiab:~4]) OR (“nutritional stimuli” [tiab:~4]) OR (“nutritional stimulus” [tiab:~4]) OR (“nutrient stimuli” [tiab:~4]) OR (“nutrient stimulus” [tiab:~4]) OR (“nutrients stimuli” [tiab:~4]) OR (“nutrients stimulus” [tiab:~4]) OR (“meal stimuli” [tiab:~4]) OR (“meal stimulus” [tiab:~4]) OR (“meals stimuli” [tiab:~4]) OR (“meals stimulus” [tiab:~4]) OR “food info*” OR “calorie info*” OR “caloric info*” OR “nutrition info*” OR “nutritional info*” OR “nutrient info*” OR “meal info*” OR “food content*” OR “calorie content*” OR “caloric content*” OR “nutrition content*” OR “nutritional content*” OR “nutrient content*” OR “meal content*” OR “visual cue” OR “visual cues” OR “food cue” OR “food cues”) AND (“diet restr*” OR “dieting restr*” OR “dietary restr*” OR “eating restr*” OR “diet*” OR “nondiet*” OR “unrestr*” OR “restr*” OR “vegan*” OR “vegetarian*” OR “ultra-process*” OR “pescatarian*” OR “low carb diet*” OR “low fat diet*” OR “low sugar diet*” OR “low calorie diet*” OR “omnivore*” OR “fast*” OR “dairy free”)

**CINAHL PLUS**

TI (Attent* OR bias* OR avoid* OR ignor* OR reject* OR desir* OR attract* OR select* OR prefer* OR engag* OR disengag* OR orient* OR gaz* OR approach* OR distract* OR focus*) OR AB (Attent* OR bias* OR avoid* OR ignor* OR reject* OR desir* OR attract* OR select* OR prefer* OR engag* OR disengag* OR orient* OR gaz* OR approach* OR distract* OR focus*)

AND TI ((“food* N4 image*”) OR (“calori* N4 image*”) OR (“nutri* N4 image*”) OR (“meal* N4 image*”) OR (“food* N4 pic*”) OR (“calori* N4 pic*”) OR (“nutri* N4 pic*”) OR (“meal* N4 pic*”) OR (“food* N4 stimul*”) OR (“calori* N4 stimul*”) OR (“nutri* N4 stimul*”) OR (“meal* N4 stimul*”) OR “food* info*” OR “calori* info*” OR “nutri* info*” OR “meal* info*” OR “food* content*” OR “calori* content*” OR “nutri* content*” OR “meal* content*” OR “visual cue*” OR “food* cue*”) OR AB ((“food* N4 image*”) OR (“calori* N4 image*”) OR (“nutri* N4 image*”) OR (“meal* N4 image*”) OR (“food* N4 pic*”) OR (“calori* N4 pic*”) OR (“nutri* N4 pic*”) OR (“meal* N4 pic*”) OR (“food* N4 stimul*”) OR (“calori* N4 stimul*”) OR (“nutri* N4 stimul*”) OR (“meal* N4 stimul*”) OR “food* info*” OR “calori* info*” OR “nutri* info*” OR “meal* info*” OR “food* content*” OR “calori* content*” OR “nutri* content*” OR “meal* content*” OR “visual cue*” OR “food* cue*”)

AND TI (“diet* restr*” OR “eat* restr*” OR diet* OR nondiet* OR unrestr* OR restr* OR vegan* OR vegetarian* OR “ultra-process*” OR pescatarian* OR “low* carb* diet*” OR “low* fat* diet*” OR “low* sugar* diet*” OR “low* calori* diet*” OR omnivore* OR fast* OR “dairy free”) OR AB (“diet* restr*” OR “eat* restr*” OR diet* OR nondiet* OR unrestr* OR restr* OR vegan* OR vegetarian* OR “ultra-process*” OR pescatarian* OR “low* carb* diet*” OR “low* fat* diet*” OR “low* sugar* diet*” OR “low* calori* diet*” OR omnivore* OR fast* OR “dairy free”)

**ProQuest Dissertations**

(Attent* OR bias* OR avoid* OR ignor* OR reject* OR desir* OR attract* OR select* OR prefer* OR engag* OR disengag* OR orient* OR gaz* OR approach* OR distract* OR focus*) AND ((“food* “NEAR/4” image*”) OR (“calori* “NEAR/4” image*”) OR (“nutri* “NEAR/4” image*”) OR (“meal* “NEAR/4” image*”) OR (“food* “NEAR/4” pic*”) OR (“calori* “NEAR/4” pic*”) OR (“nutri* “NEAR/4” pic*”) OR (“meal* “NEAR/4” pic*”) OR (“food* “NEAR/4” stimul*”) OR (“calori* “NEAR/4” stimul*”) OR (“nutri* “NEAR/4” stimul*”) OR (“meal* “NEAR/4” stimul*”) OR “food* info*” OR “calori* info*” OR “nutri* info*” OR “meal* info*” OR “food* content*” OR “calori* content*” OR “nutri* content*” OR “meal* content*” OR “visual cue*” OR “food* cue*”) AND (“diet* restr*” OR “eat* restr*” OR diet* OR nondiet* OR unrestr* OR restr* OR vegan* OR vegetarian* OR “ultra-process*” OR pescatarian* OR “low* carb* diet*” OR “low* fat* diet*” OR “low* sugar* diet*” OR “low* calori* diet*” OR omnivore* OR fast* OR “dairy free”)

**SocArXiv Papers**

Attent* | bias* | avoid* | ignor* | reject* | desir* | attract* | select* | prefer* | engag* | disengag* | orient* | gaz* | approach* | distract* | focus* AND food | calori* AND restr* | diet*

The proximity operator in our search strategy was modified to comply with each database.

**CMA Multivariate Method**

To include multiple outcomes from the same study, CMA software requires the creation of a mean composite effect size of these outcomes and a calculation of the variance of this effect size using the correlations between each outcome.

Due to the nature of our attentional bias outcomes, e.g. some indices can show opposite effects, creating a mean composite averaged out some of these effects and mask important distinctions between attentional mechanisms. Thus, we opted for a univariate analysis to capture information on one select attentional mechanism from each study.

**Selected Effect Sizes**

The following effect sizes were included as attentional maintenance:

1. Total dwell time during instructed viewing (Werthmann et al, 2013; Werthmann et al, 2013; Werthmann et al, 2014; Liu et al, 2021; Nannt et al, 2025)
2. Total dwell time during free-viewing (Garcia-Burgos et al, 2017; Hummel et al, 2018; Xu et al, 2023)
3. Total fixation duration during free-viewing (Chen et al, 2023)
4. Total fixation duration during instructed viewing (Van Ens et al, 2019)
5. RT on 1000ms trials (Dondzilo et al, 2022, Husted et al, 2016; Kim et al, 2014)
6. RT on 1500ms trials (Meule et al, 2012; Sambal et al, 2021)
7. RT on 500ms trials (Ahern et al, 2010; Hardman et al, 2013; Freijy et al, 2014; Veenstra et al, 2010; Jiang et al, 2024 (478ms))
8. RT on 2000ms trials (Wilson and Wallis, 2013 (three samples); Werthmann et al, 2011)
9. RT on mix of 500ms and 2000ms trials (Brignell et al, 2009)

*For Husted et al (2016), Sambal et al (2021) and Meule et al (2012), we used overall RT during the flanker task as maintenance, since data was excluded below 150ms and above 1000ms (Husted et al, 2016), below 200ms and above 1500ms (Sambal et al, 2021) and below 150ms (Meule et al, 2012).

The following effect sizes were included as attentional orienting:

1. Gaze direction/first fixation location during free-viewing (Graham et al, 2011; Garcia-Burgos et al 2017; Chen et al, 2023)
2. Gaze direction/first fixation location during instructed-viewing (Werthmann et al, 2013; Werthmann et al, 2013; Werthmann et al, 2014; Nannt et al, 2025)
3. First fixation duration during instructed viewing (Liu et al, 2021)
4. Time to first fixation during free-viewing (Xu et al, 2023)
5. Percentage accuracy on 118ms trials; Food vs non-food T1 enhances attentional blink on T2 (Neimeijer et al, 2013; Kirsten et al, 2019; Jiang et al, 2024)
6. RT on “engagement match” trials (Donofry et al, 2019; although trials were not limited to 200ms, each trial included scanning and orienting to the correct target)
7. RT on 200ms trials (Wilson and Wallis, 2013; three samples)
8. RT on “switching” task (Dondzilo et al, 2022)

**Description of samples**

The 29 included samples had a total of 1727 participants, who were mainly adult females with a healthy BMI; there were 18 female only (Nannt et al, 2025; Van Ens et al, 2019; Kim et al, 2014; Ahern et al, 2010; Graham et al, 2011; Donofry et al, 2019; Sambal et al, 2021; Neimeijer et al, 2013; Veenstra et al, 2010; Wilson and Wallis, 2013 (three samples); Werthmann et al, 2011; Werthmann et al, 2013; Werthmann et al, 2013; Werthmann et al, 2014; Muele et al, 2012; Liu et al, 2021) and 11 mixed gender samples (Kirsten et al, 2019; Husted et al, 2016; Hardman et al, 2013; Brignell et al, 2009; Chen et al, 2023; Hummel et al, 2018; Xu et al, 2023; Jiang et al, 2024; Dondzilo et al, 2022; Garcia-Burgos et al, 2017; Freijy et al, 2014) with a mean age range from 19-34 years, and only two samples had a mean BMI in the overweight category (Graham et al, 2011; Brignell et al, 2009). All studies that provided recruitment information included students and/or included recruitment from universities. Two studies did not provide any recruitment information (Chen et al, 2023; Werthmann et al, 2013). Eleven of the 29 samples were screened for clinical EDs (Nannt et al, 2025; Wilson and Wallis, 2013 (three samples); Jiang et al, 2024; Garcia-Burgos et al, 2017; Sambal et al, 2021; Kirsten et al, 2019; Husted et al, 2016; Kim et al, 2014; Van Ens et al, 2019). The most common restraint measure was the DEBQ, used in 14 samples (Husted et al, 2016; Kirsten et al, 2019; Ahern et al, 2010; Brignell et al, 2009; Chen et al, 2023; Hummel et al, 2018; Sambal et al, 2021; Jiang et al, 2024; Veenstra et al, 2010; Freijy et al, 2014; Wilson and Wallis, 2013 (three samples); Garcia-Bugos et al, 2017), followed by the RS used in 12 samples (Liu et al, 2021; Werthmann et al, 2011; Werthmann et al, 2013; Werthmann et al, 2013; Werthmann et al, 2014; Muele et al, 2012; Dondzilo et al, 2022; Neimeijer et al, 2013; Donofry et al, 2019; Xu et al, 2023; Graham et al, 2011; Van Ens et al, 2019). Two studies used the EDEQ (Nannt et al, 2025; Kim et al, 2014) and one study used the TFEQ (Hardman et al, 2013).

**Attentional Maintenance additional figures**

**Figure 1. Meta-analysis of associations between attentional maintenance and dietary restraint (K=25)**

**
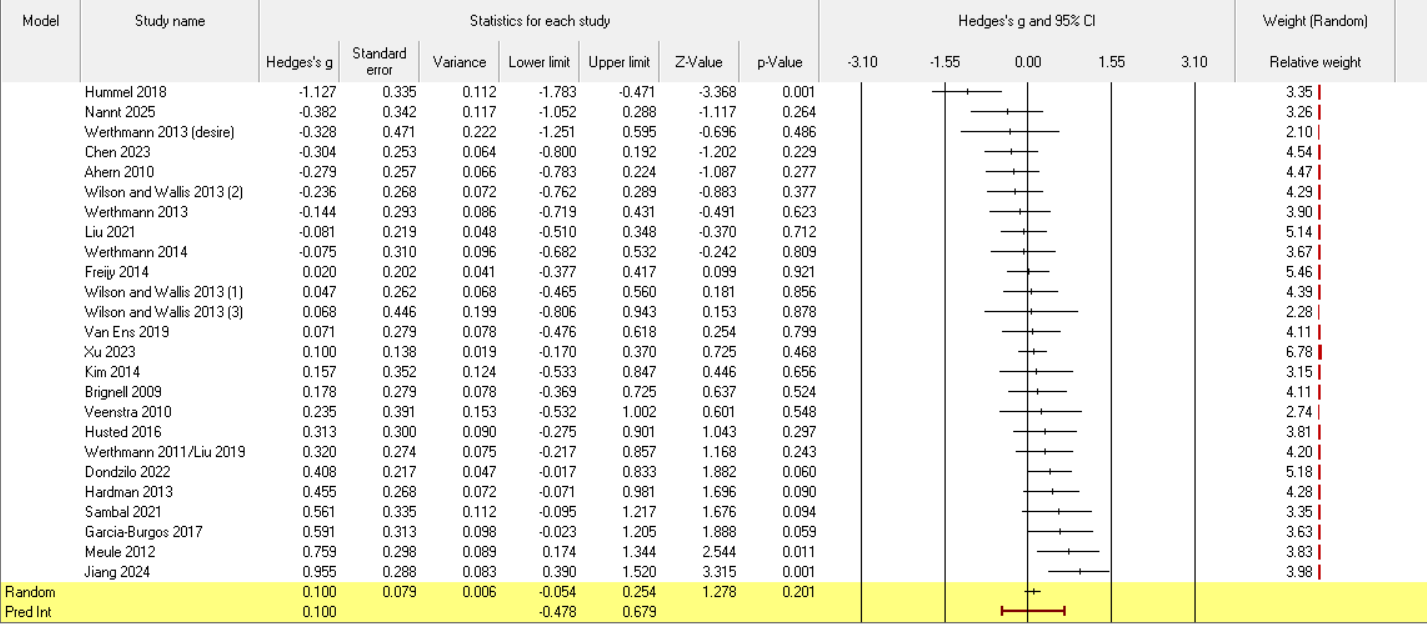
**

**Figure 2. Meta-analysis of associations between attentional maintenance and dietary restraint in studies using eye-tracking (K=10)**


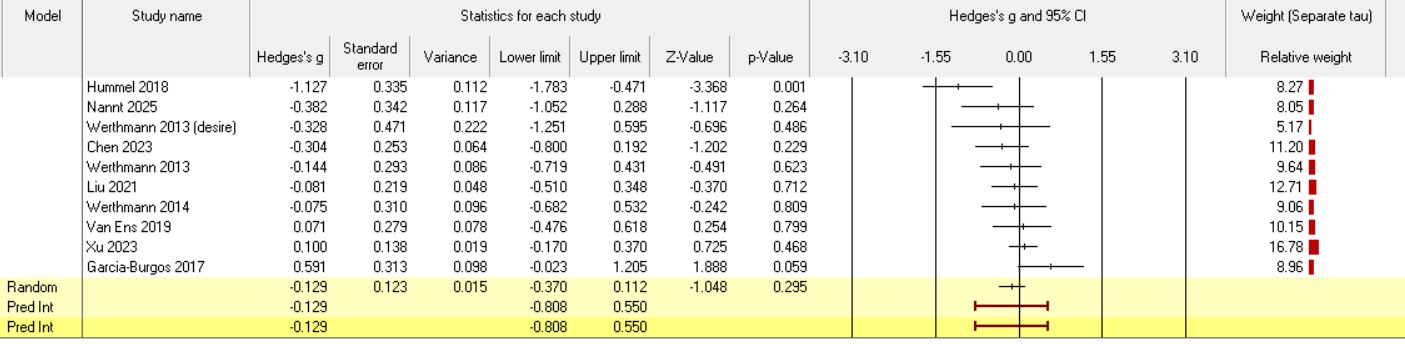


**Figure 3. Meta-analysis of associations between attentional maintenance and dietary restraint in studies using free-viewing (K=4)**


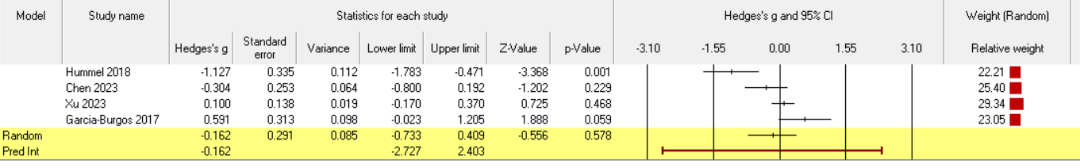


**Figure 4. Meta-analysis of associations between attentional maintenance and dietary restraint in studies using instructed-viewing (K=6)**


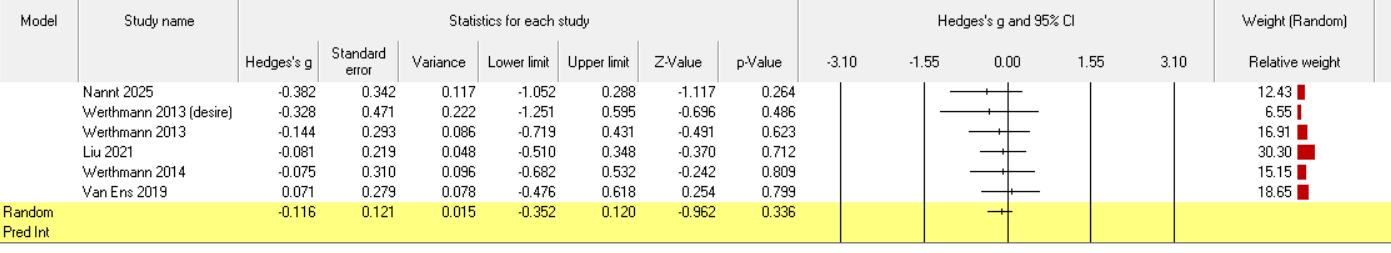


**Figure 5. Meta-analysis of associations between attentional maintenance and dietary restraint in studies using the dot probe task (K=15)**


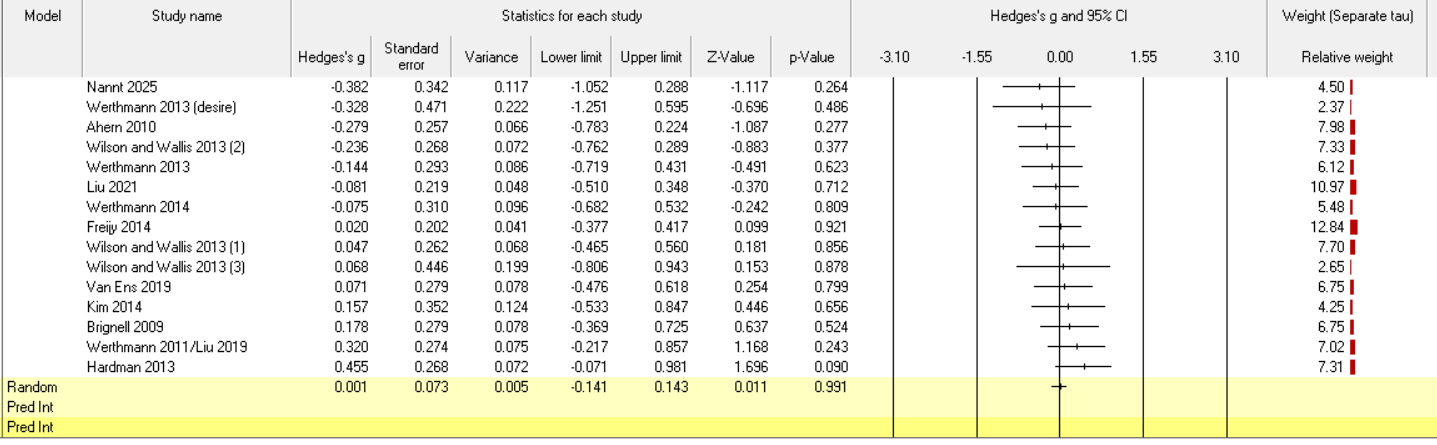


**Figure 6. Meta-analysis of associations between attentional maintenance and dietary restraint in studies using irrelevant food stimuli (K=16)**


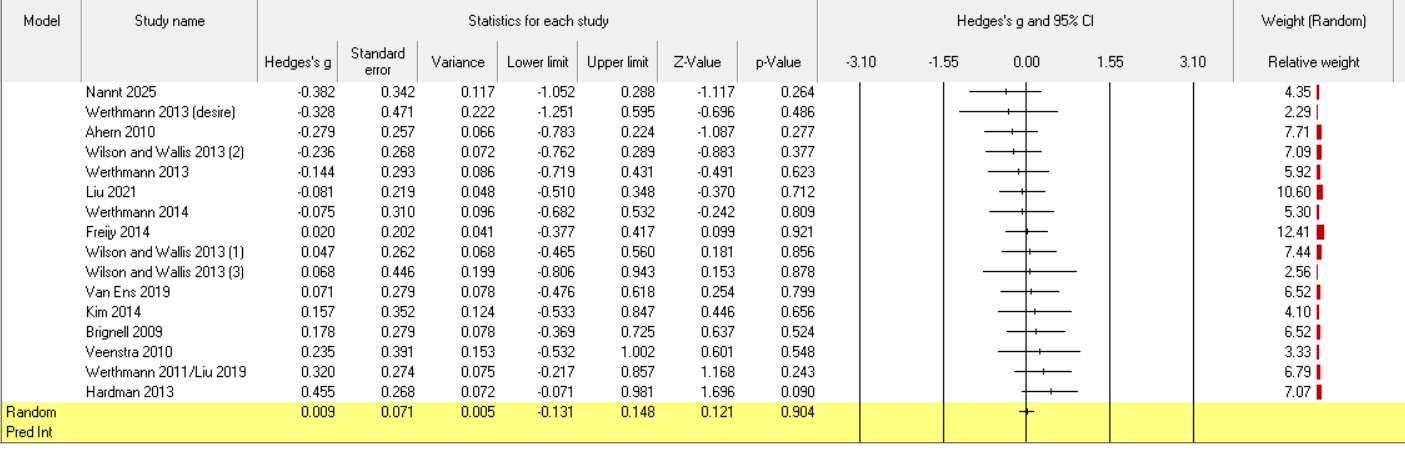


**Figure 7. Meta-analysis of associations between attentional maintenance and dietary restraint in studies using HC stimuli (K=19)**

**
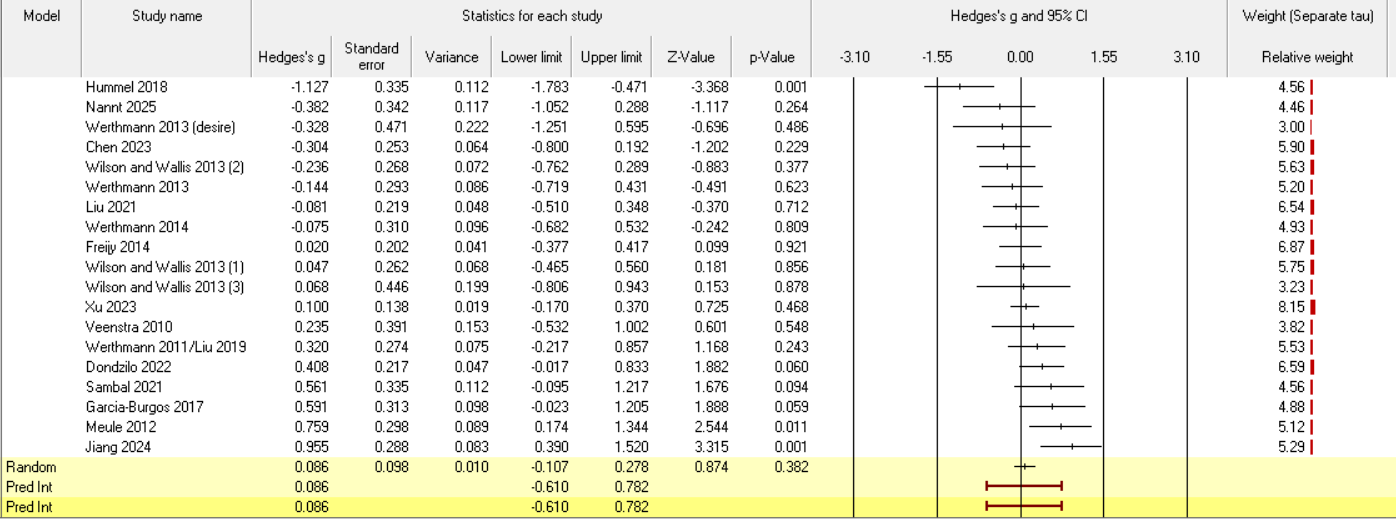
**

**Figure 8. Meta-analysis of associations between attentional maintenance and dietary restraint in studies using mixed food stimuli (K=6)**


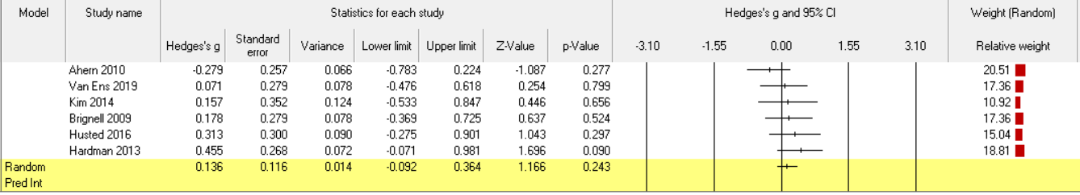


**Attentional Orienting Results and Figures**

**Experimental Paradigm**

We compared all *response tasks* to *all viewing* paradigms (instructed and free-viewing). Contrary to predictions, we found no significant associations in *response tasks*(HG = 0.089 [-0.211, 0.389] P =0.560) nor for *viewing* paradigms (HG = -0.049 [-0.289, 0.191] P=0.688). The difference between these effect sizes was not significant (Q= 0.499 P=0.480). Heterogeneity was moderate-high for *response tasks*  (Q= 20.142 P=0.005 I2=65.25) and moderate for *viewing* paradigms (Q= 16.46 P=0.058 I2=45.32%).

In the unadjusted meta-regression model, the *experimental paradigm*coefficient was not significant (B= 0.146 [-0.234,0.526], R2= -0.14; Q=0.57 P=0.451). Heterogeneity was 56.3%.

Adjusting for *clinical ED screening* and *type of restraint scale* did not change the significance: B= 0.224 [-0.193, 0.642] P=0.292 R2= -0.12 Q=2.44 P=0.655 I2=55.9%.

**Figure 9. Meta-analysis of associations between restraint and orienting using response tasks (K=8)**


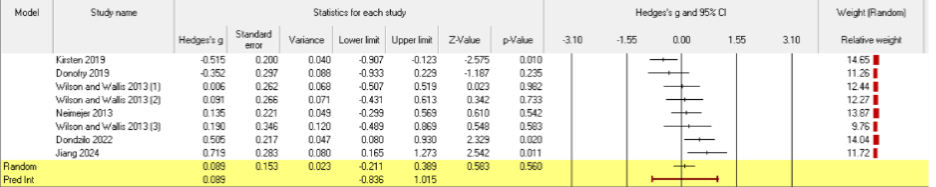


**Figure 10. Meta-analysis of associations between restraint and orienting using eye-tracking (k=10)**


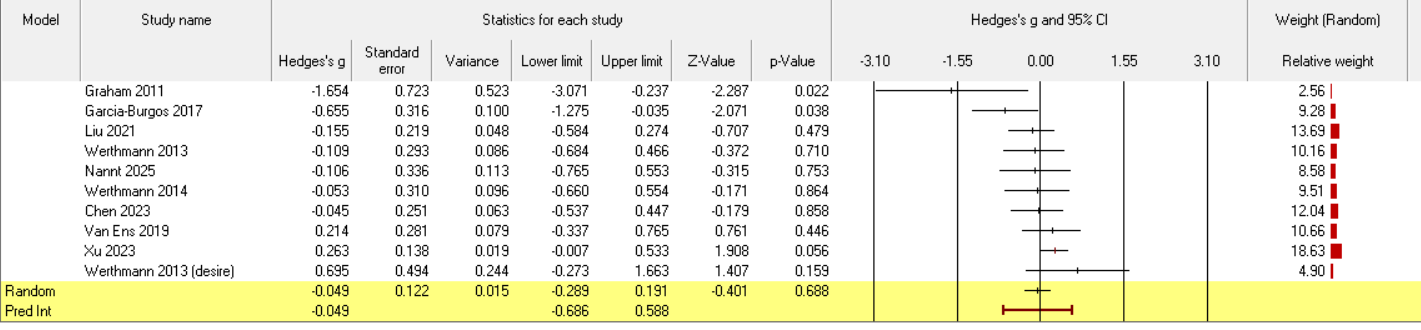


**Figure 11. Meta-analysis of associations between restraint and orienting using free-viewing (K=4)**


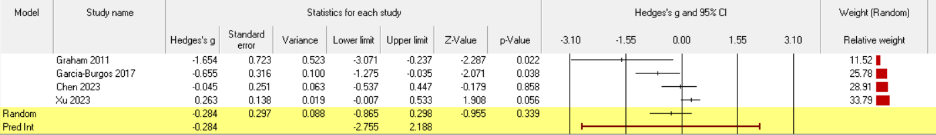


**Figure 12. Meta-analysis of associations between restraint and orienting using instructed-viewing (k=6)**


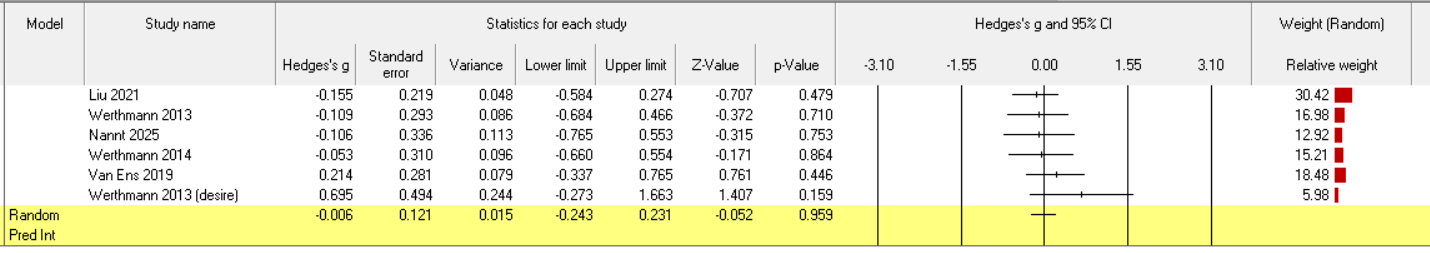


 We sub-grouped eye-tracking studies into *instructed viewing* and *free-viewing*to explore differences between these paradigms. We found no significant associations between restraint and ABs among *instructed viewing* studies (HG =0.006 [-0.243, 0.231] P=0.959; Q=3.324 P=0.650 i2=0%) nor among*free-viewing* studies (HG = -0.284 [-0.865, 0.298] P=0.339; Q=13.05 P=0.005 I2=77.01%). Comparisons between all three effect sizes for *response tasks, instructed-viewing* and *free-viewing* were not statistically significant: Q=1.255, P=0.534. Given the high heterogeneity in *free-viewing* paradigms , we did not include this subgroup in  further analyses.

Comparisons between*instructed viewing* and *response task* effect sizes did not reach significance: Q=0.240 P=0.624.

In the unadjusted meta-regression, the *experimental paradigm*coefficient was not significant B=0.065 [-0.348, 0.479] R2= -0.17 Q=0.10 P=0.757 I2=48.9%, (45.2% in null model. ). We adjusted for *clinical ED screening* only due to the low study number in the *instructed viewing*group and this did not change significance: B= 0.100 [-0.308, 0.507] P=0.633 R2= -0.05; Q=1.21, P=0.544 I2=45.8%.

**Response Task Type**

We found no associations between restraint and orienting among*dot probe* studies (HG=0.024 [-0.167, 0.215] P=0.806) and nor among the*other tasks* group (HG = 0.093 [-0.374, 0.561] P=0.696), with the difference between these effect sizes also non-significant: Q=0.072 P=0.788  Heterogeneity was low in the *dot probe* group (Q=3.684, P=0.884  I2=0%) and high in the *other tasks* group (Q=19.95 P=0.001 I2=79.95).

Given the high heterogeneity in the *other tasks* group, we did not run any further analyses.

**Figure 13. Meta-analysis of associations between restraint and orienting using dot probe task (k=9)**


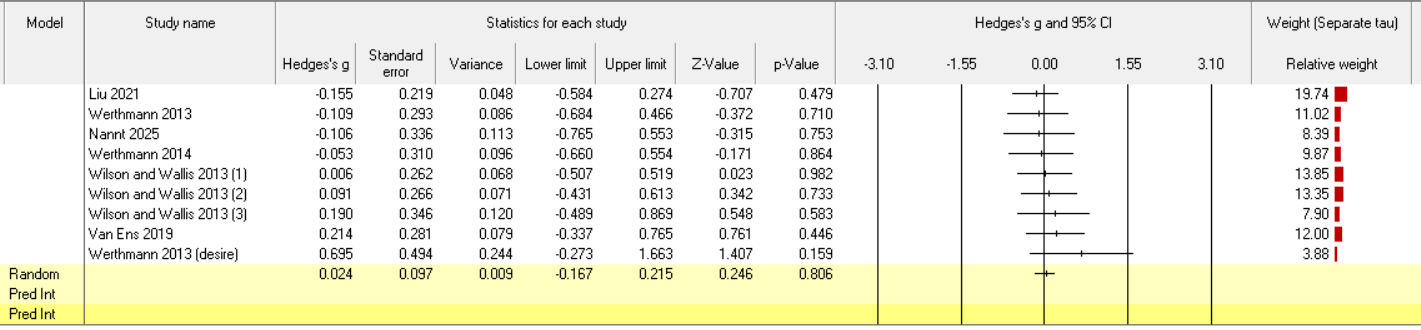


**Figure 14. Meta-analysis of associations between restraint and orienting using alternative tasks (K=5)**


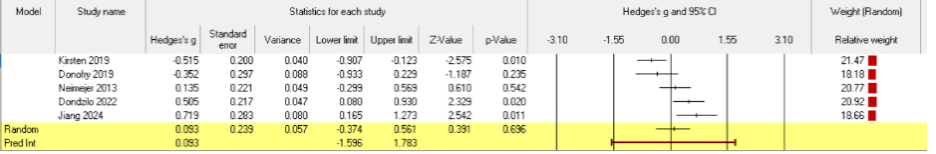


**Food Stimuli Relevance**

We found no significant associations among *irrelevant* stimuli: HG=-0.013 [-0.194,  0.169] P=0.891,  nor among *relevant*stimuli: HG=0.194 [-0.348, 0.736]P=0.483. The difference between effect sizes was non-significant Q=0.502 P=0.479 Heterogeneity was low in the *irrelevant* group: Q=5.134 P=0.822 I2=0, but high in the *relevant* group Q=17.724 P=0.001 I2=83.074 so we did not run any further analyses.

**Figure 15. Meta-analysis of associations between restraint and orienting using irrelevant stimuli (k=10)**

**
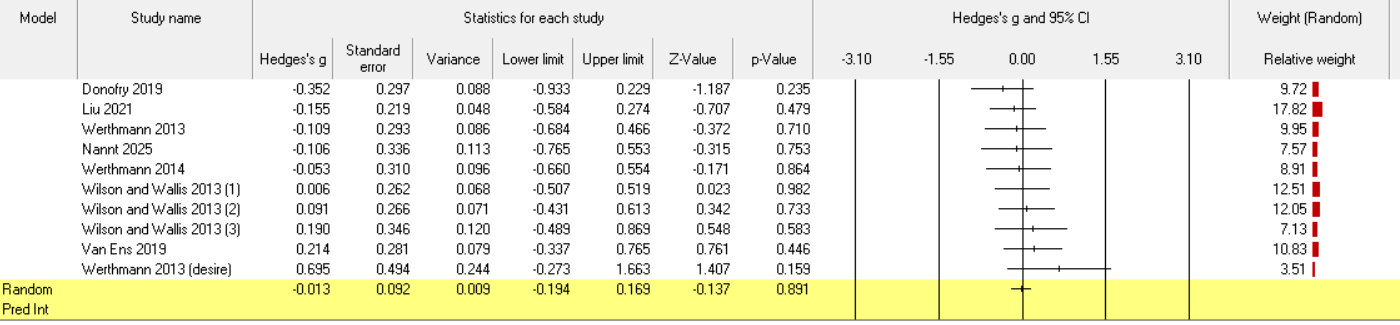
**

**Figure 16. Meta-analysis of associations between restraint and orienting relevant stimuli (k=4)**


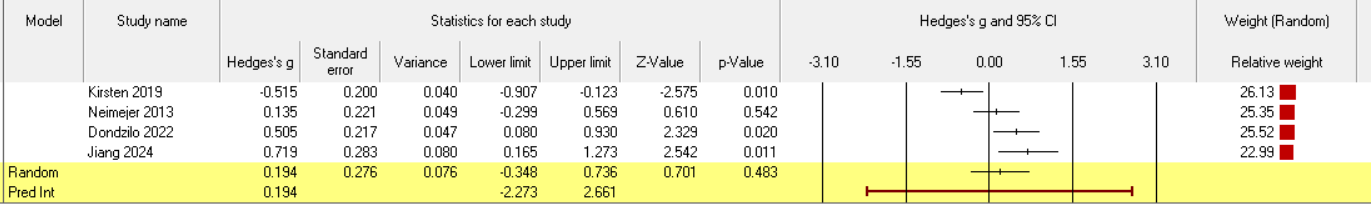


**Type of Target Food Stimuli**

As we only had two studies in the *mixed food l*evel of our stimuli type variable, we did not run any statistical analyses for this group.

**Figure 17. Meta-analysis of associations between restraint and orienting using HC stimuli (K=16)**

**
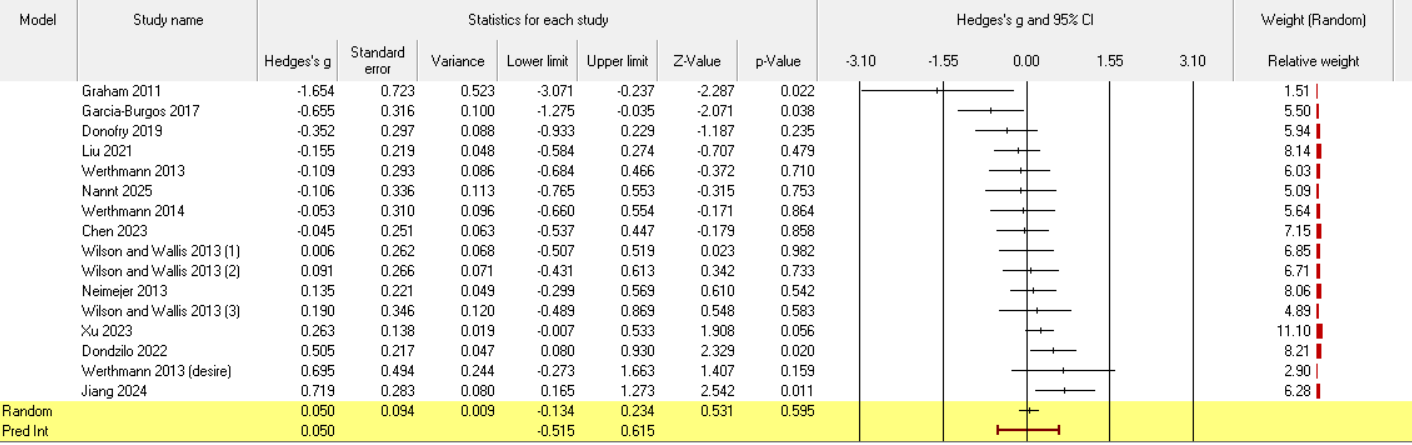
**

 HG= 0.050 [-0.134,  0.234] P=0.595; Q=28.052, P=0.021 I²=46.53%

**Attentional biases and ED Symptoms Figures**

**Figure 18. Meta-analysis of associations between maintenance and ED symptoms using the EDEQ (K=4)**


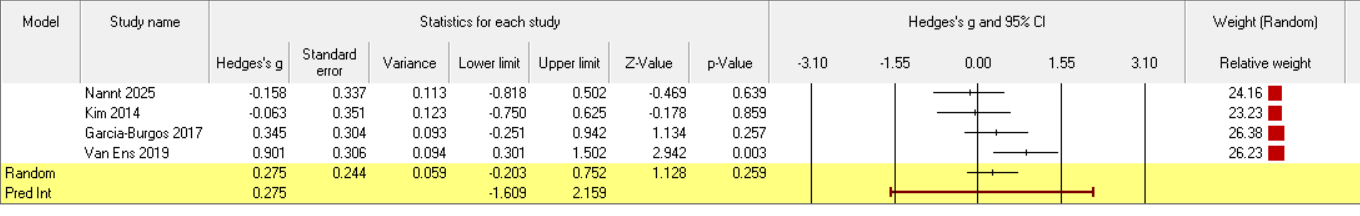


**Figure 19. Meta-analysis of associations between maintenance and ED symptoms using the EDI-2 (K=3)**


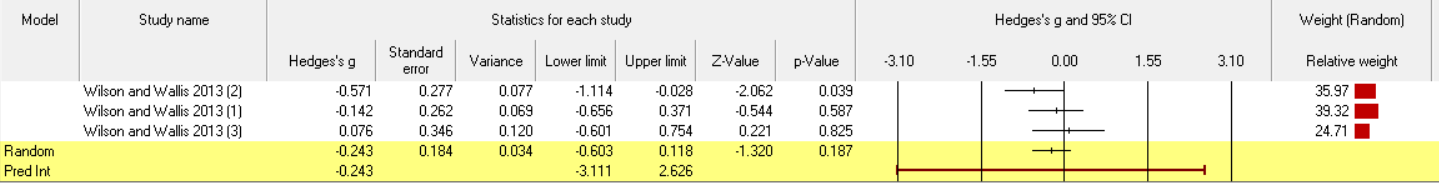


**Figure 20. Meta-analysis of associations between maintenance and ED symptoms using different outcome measures (K=3)**


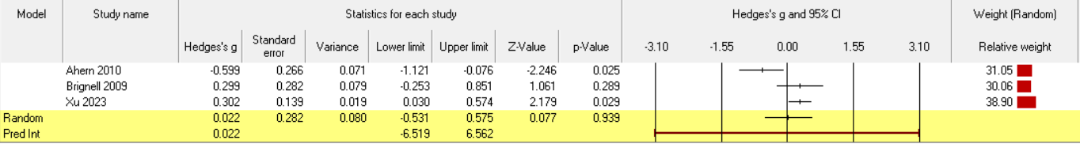


**Publication Bias Egger’s regression test**

**Figure 21. Funnel plot of all included samples, P=0.967**


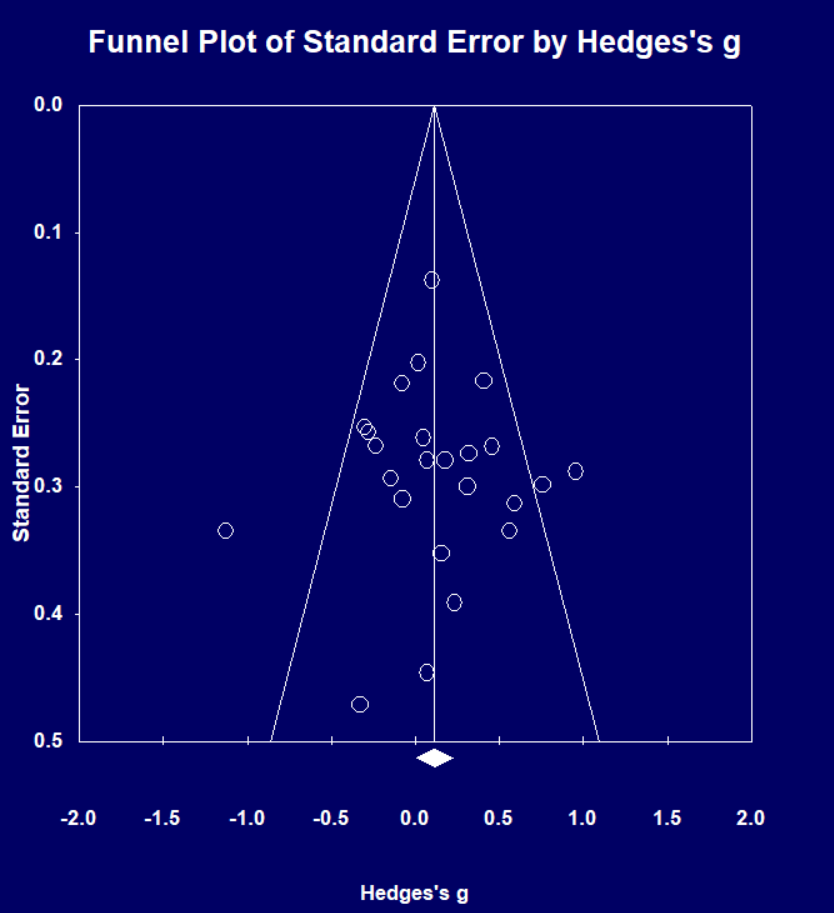


**Figure 22. Funnel plot of Response Tasks, P=0.507**


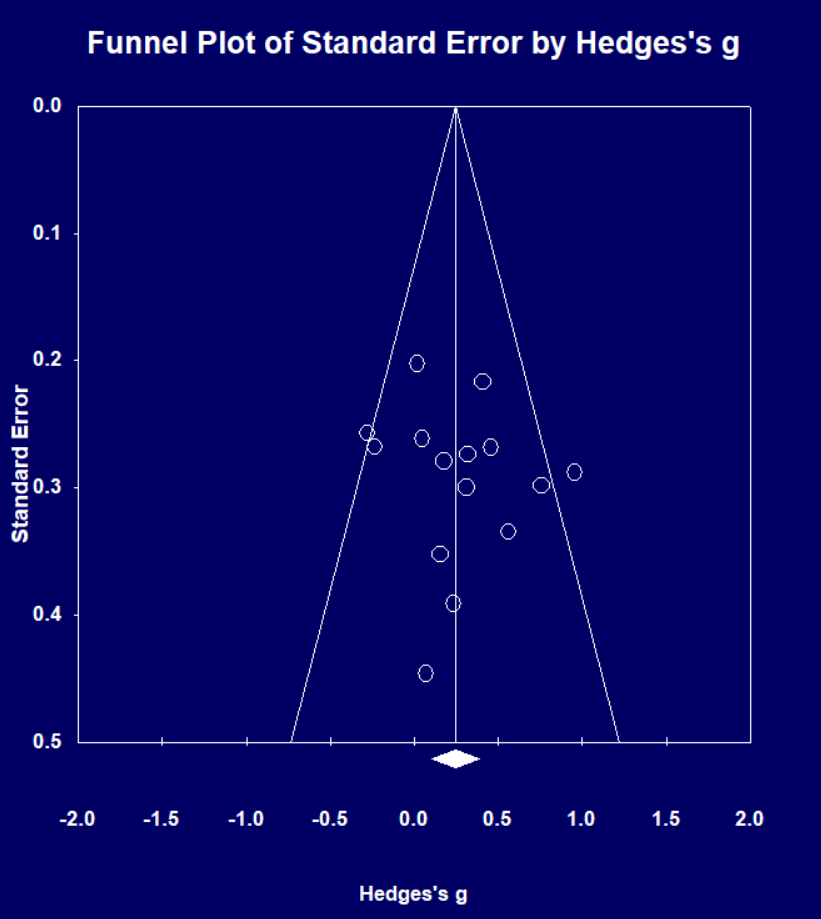


**Figure 23. Funnel plot of Instructed-viewing paradigms, P=0.320**


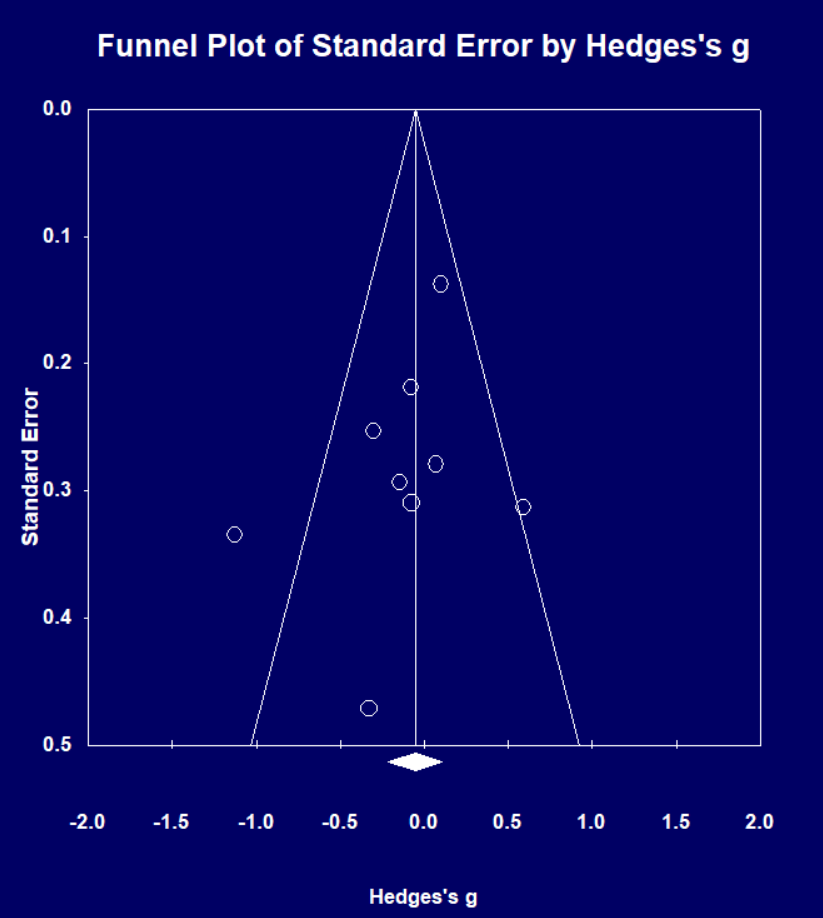


**Figure 24. Funnel plot of Free-viewing paradigms, P=0.324**


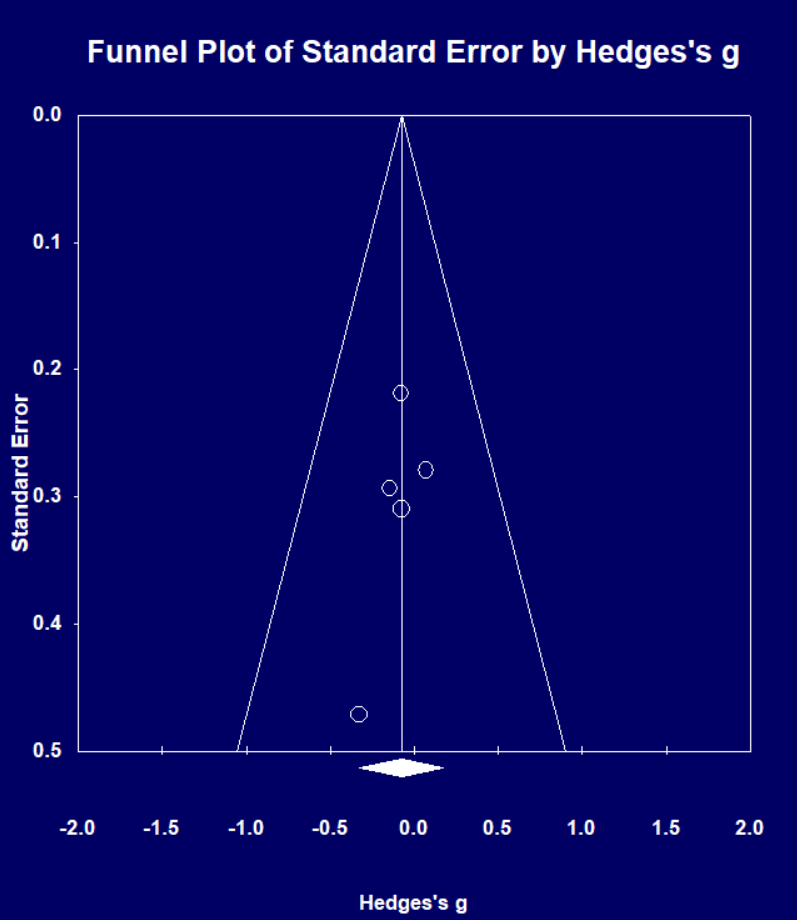


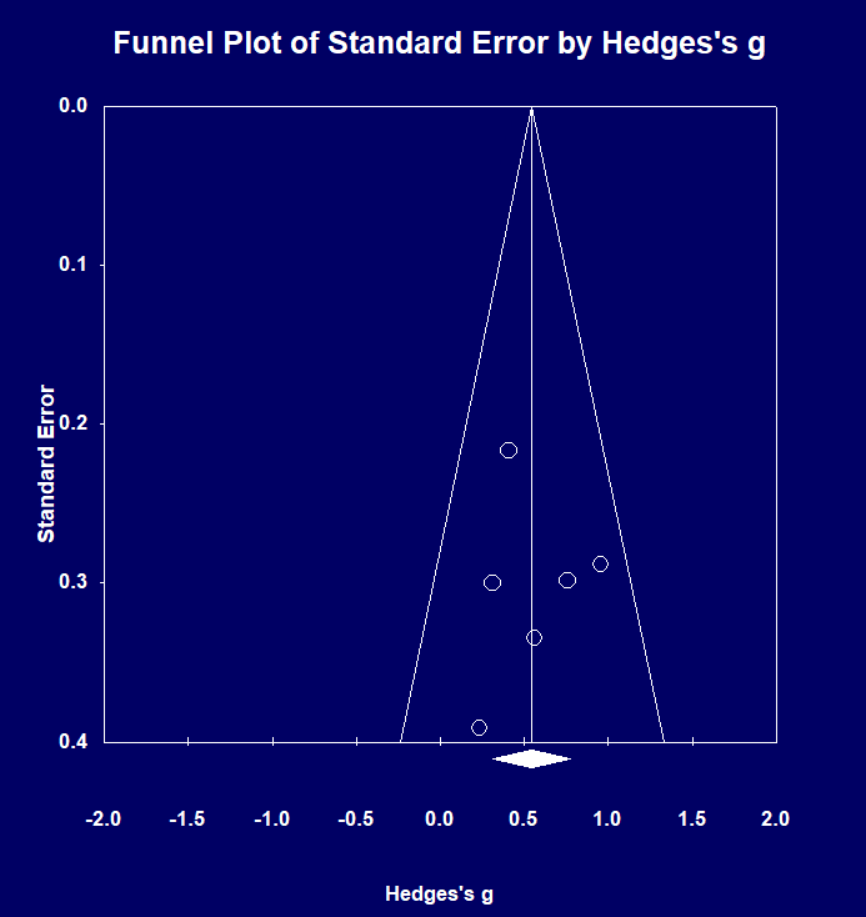
**Figure 25. Funnel plot of Dot probe studies, P=0.918**


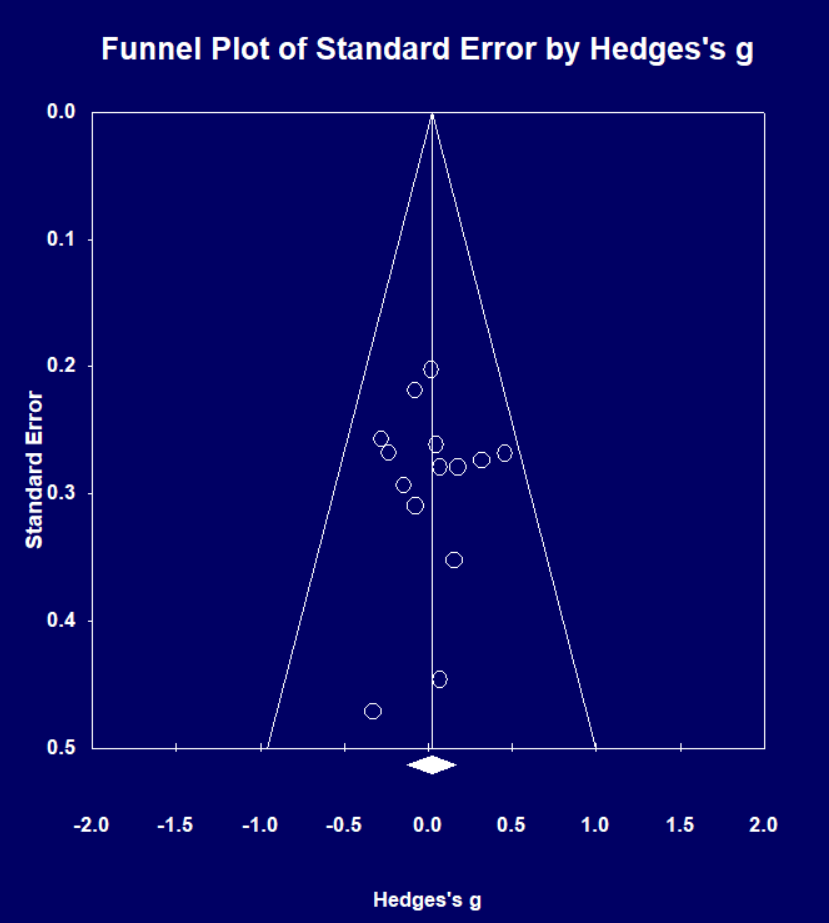
**Figure 26. Funnel plot of alternative task studies, P=0.999**


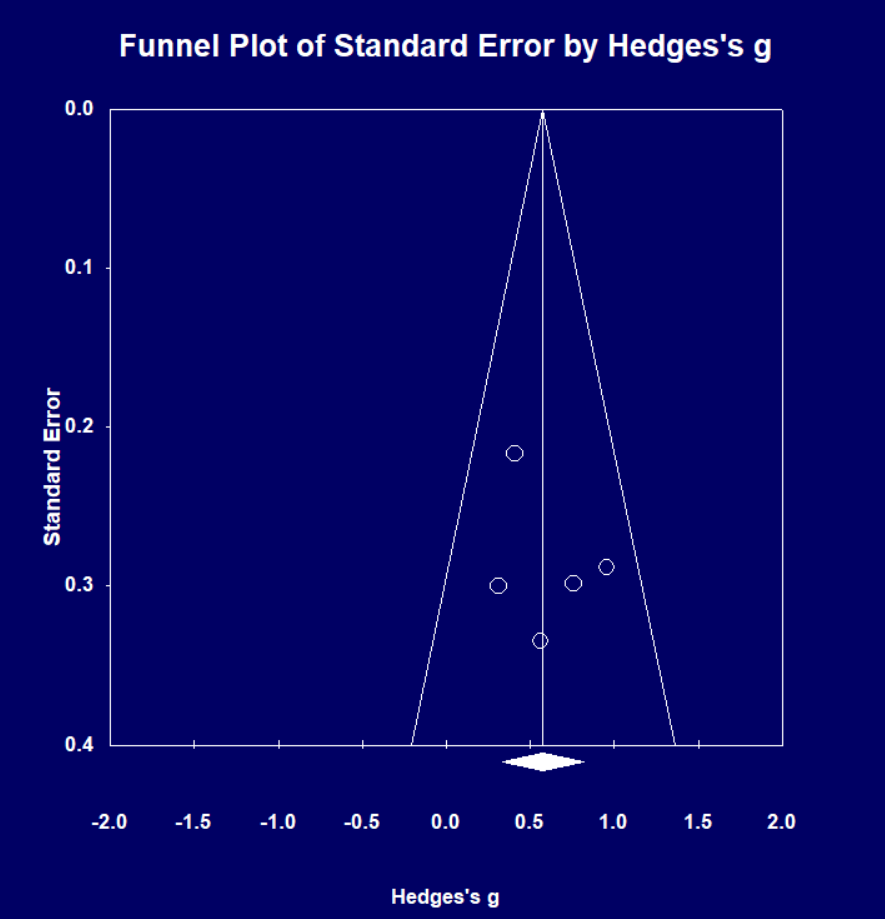
**Figure 27. Funnel plot of relevant stimuli, P=0.546**

**Figure 28. Funnel plot of Irrelevant stimuli, P=0.882**


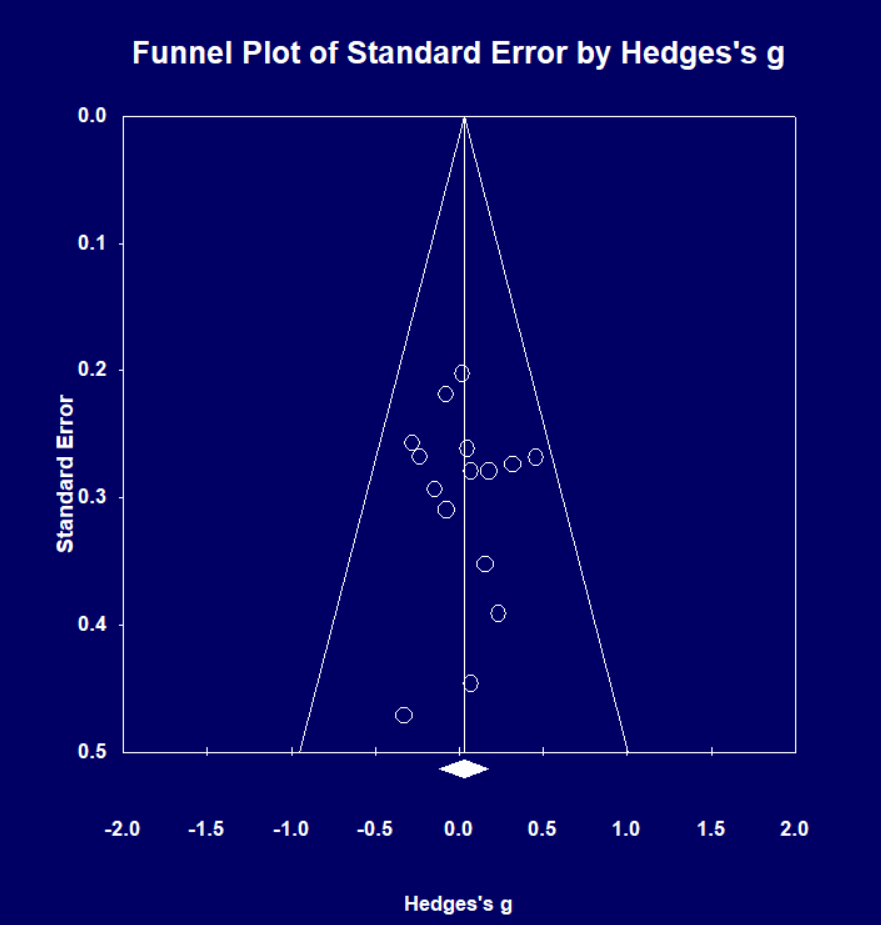


**Figure 29. Funnel plot of HC stimuli, P=0.952**


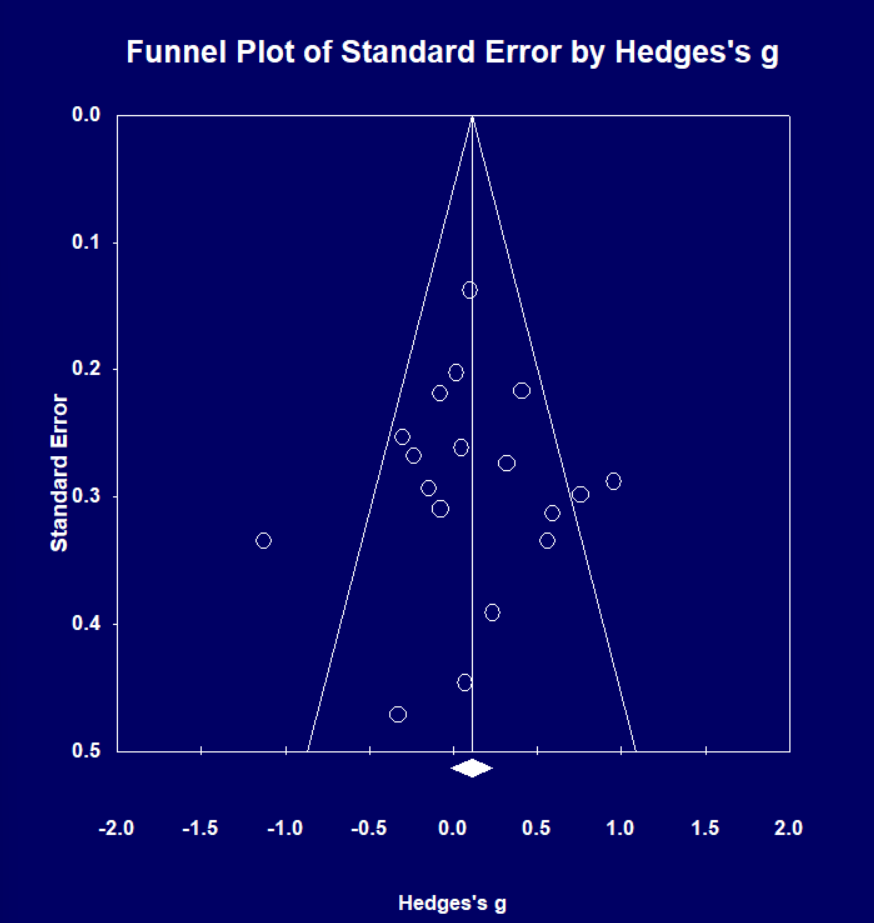


**Figure 30. Funnel plot of Mixed food stimuli, P=0.573**


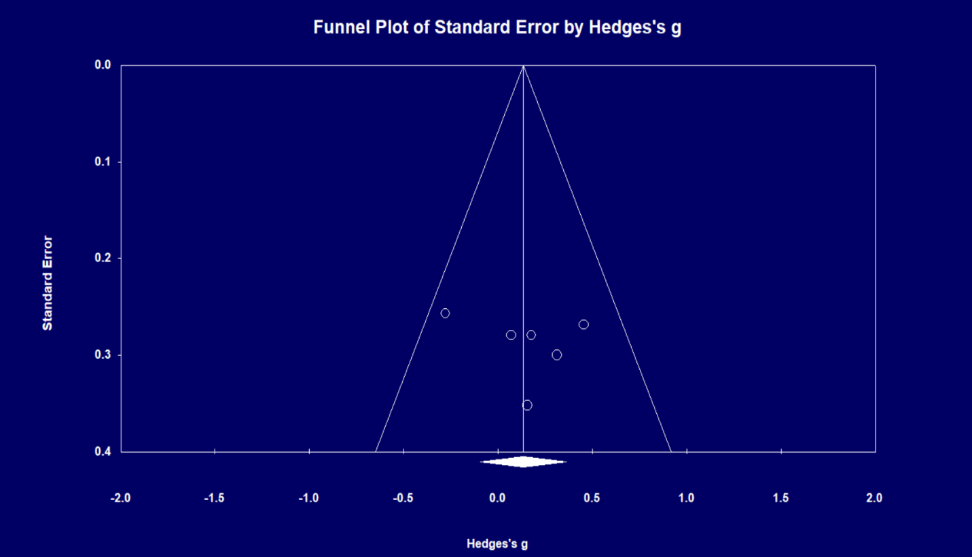

Supplement: Supplementary file 3 — Figure S1: Meta‐analysis of associations between attentional maintenance and dietary restraint (K = 25). Figure S2: Meta‐analysis of associations between attentional maintenance and dietary restraint in studies using eye‐tracking (K = 10). Figure S3: Meta‐analysis of associations between attentional maintenance and dietary restraint in studies using free‐viewing (K = 4). Figure S4: Meta‐analysis of associations between attentional maintenance and dietary restraint in studies using instructed‐viewing (K = 6). Figure S5: Meta‐analysis of associations between attentional maintenance and dietary restraint in studies using the dot probe task (K = 15). Figure S6: Meta‐analysis of associations between attentional maintenance and dietary restraint in studies using irrelevant food stimuli (K = 16). Figure S7: Meta‐analysis of associations between attentional maintenance and dietary restraint in studies using HC stimuli (K = 19). Figure S8: Meta‐analysis of associations between attentional maintenance and dietary restraint in studies using mixed food stimuli (K = 6). Figure S9: Meta‐analysis of associations between restraint and orienting using response tasks (K = 8). Figure S10: Meta‐analysis of associations between restraint and orienting using eye‐tracking (k = 10). Figure S11: Meta‐analysis of associations between restraint and orienting using free‐viewing (K = 4). Figure S12: Meta‐analysis of associations between restraint and orienting using instructed‐viewing (k = 6). Figure S13: Meta‐analysis of associations between restraint and orienting using dot probe task (k = 9). Figure S14: Meta‐analysis of associations between restraint and orienting using alternative tasks (K = 5). Figure S15: Meta‐analysis of associations between restraint and orienting using irrelevant stimuli (k = 10). Figure S16: Meta‐analysis of associations between restraint and orienting relevant stimuli (k = 4). Figure S17: Meta‐analysis of associations between restraint and orienting using HC stimuli [file EAT-59-1426-s003.docx]
